# PGAM1 Inhibition Promotes HCC Ferroptosis and Synergizes with Anti‐PD‐1 Immunotherapy

**DOI:** 10.1002/advs.202301928

**Published:** 2023-09-14

**Authors:** Yimin Zheng, Yining Wang, Zhou Lu, Jinkai Wan, Lulu Jiang, Danjun Song, Chuanyuan Wei, Chao Gao, Guoming Shi, Jian Zhou, Jia Fan, Aiwu Ke, Lu Zhou, Jiabin Cai

**Affiliations:** ^1^ Department of Liver Surgery and Transplantation Liver Cancer Institute Zhongshan Hospital Fudan University; Key Laboratory of Carcinogenesis and Cancer Invasion Shanghai Key Laboratory of Organ Transplantation Zhongshan Hospital Shanghai 200032 P. R. China; ^2^ Shanghai Key Laboratory of Medical Epigenetics International Co‐laboratory of Medical Epigenetics and Metabolism Ministry of Science and Technology Institutes of Biomedical Sciences Fudan University Shanghai 200032 P. R. China; ^3^ Department of Medicinal Chemistry School of Pharmacy Fudan University Shanghai 201203 P. R. China; ^4^ Department of Interventional Therapy The Cancer Hospital of the University of Chinese Academy of Sciences (Zhejiang Cancer Hospital) Institute of Basic Medicine and Cancer (IBMC) Chinese Academy of Sciences Hangzhou Zhejiang 310022 P. R. China

**Keywords:** carcinogen metabolism, ferroptosis, hepatocellular carcinoma, immunotherapy

## Abstract

The combination of immunotherapy and molecular targeted therapy exhibits promising therapeutic efficacy in hepatocellular carcinoma (HCC), but the underlying mechanism is still unclear. Here, phosphoglycerate mutase 1 (PGAM1) is identified as a novel immunometabolic target by using a bioinformatic algorithm based on multiple HCC datasets. PGAM1 is highly expressed in HCC and associated with a poor prognosis and a poor response to immunotherapy. In vitro and in vivo experiments indicate that targeting PGAM1 inhibited HCC cell growth and promoted the infiltration of CD8^+^ T‐cells due to decreased enzymatic activity. Mechanistically, inhibition of PGAM1 promotes HCC cell ferroptosis by downregulating Lipocalin (LCN2) by inducing energy stress and ROS‐dependent AKT inhibition, which can also downregulate Programmed death 1‐ligand 1 (PD‐L1). Moreover, an allosteric PGAM1 inhibitor (KH3) exhibits good antitumor effects in patient‐derived xenograft (PDX) models and enhanced the efficacy of anti‐PD‐1 immunotherapy in subcutaneous and orthotopic HCC models. Taken together, the findings demonstrate that PGAM1 inhibition exerts an antitumor effect by promoting ferroptosis and CD8^+^ T‐cell infiltration and can synergize with anti‐PD‐1 immunotherapy in HCC. Targeting PGAM1 can be a promising new strategy of “killing two birds with one stone” for HCC treatment.

## Graphical abstract

Phosphoglycerate mutase 1 (PGAM1) inhibition can promote ferroptosis and bolster anti‐tumor immunity in hepatocellular carcinoma (HCC). Mechanistically, targeting PGAM1 restrains HCC growth by suppressing LCN2 and PD‐L1 expression via energy stress/ROS‐dependent inhibition of AKT and potentiates robust CD8^+^ T‐cell infiltration. PGAM1 can serve as a novel potential immunometabolic target and synergize with anti‐PD‐1 immunotherapy for HCC patients.

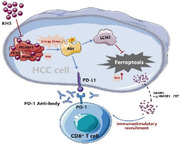

## 1 Introduction

Liver cancer was the sixth most common cancer, with 905 677 new cases, and the third deadliest malignancy, with 830 180 new deaths worldwide in 2020;^[^ ^1^ ^]^ 75%–85% are liver cancers are hepatocellular carcinoma (HCC).^[^ ^2^ ^]^ Tumor cells are capable of metabolic reprogramming and development of an immunosuppressive microenvironment to facilitate their survival. Selecting one target with dual effects in restraining HCC cell growth and simultaneously potentiating antitumor immunity seems to be a reasonable strategy. Recently, clinical trials of immune checkpoint inhibitors (ICIs), exemplified by anti‐programmed death 1 (PD‐1) antibodies, have demonstrated unprecedented responses in some HCC patients.^[^ ^3^ ^]^ However, only a limited number of patients are responsive to immunotherapies, and no biomarker to predict response is available in HCC. Immunotherapy responsiveness can be attributed to various factors, including aberrant metabolism of tumor cells and limited infiltration of effector T‐cells. Thus, there is an urgent clinical need to improve ICI immunotherapeutic efficacy and identify novel combination regimens for HCC patients. A thorough exploration of aberrant metabolism and immune suppression in HCC has been a hotspot issue.

Phosphoglycerate mutase 1 (PGAM1) is aberrantly overexpressed in various human cancers, including HCC^[^ ^4^ ^]^ and plays a crucial role in cancer metabolism and tumor progression via its metabolic mechanism.^[^ ^5^ ^]^ PGAM1 is primarily considered a glycolytic enzyme that catalyzes the conversion of 3‐phosphoglycerate (3‐PG) to 2‐phosphoglycerate (2‐PG) and coordinating biosynthesis pathways, including the pentose phosphate pathway and serine synthesis pathway. In addition to glycolysis regulation, other carcinogenic effects of PGAM1 have been recently recognized and explored. For instance, PGAM1 could facilitate homologous recombination in DNA repair by metabolically dependent regulation of the dNTP pool.^[^ ^6^ ^]^ Apart from this metabolic function, PGAM1 could also promote cancer cell migration through interaction with alpha‐smooth muscle actin (ACTA2) independent of its canonical enzymatic activity.^[^ ^7^ ^]^

The present study shows that high PGAM1 expression correlates with a poor prognosis in HCC patients and attenuates the infiltration and activation of CD8^+^ T‐cells. Intriguingly, inhibition of PGAM1 could promote HCC ferroptosis by suppressing lipocalin 2 (LCN2), which encodes an iron‐sequestering cytokine. Recently, substantial progress has been made in elucidating how oncogenic pathways and metabolic reprogramming confer sensitivity to ferroptosis in tumor cells. We first identified that PGAM1 inhibition could reduce LCN2 expression via energy stress/ROS‐dependent inhibition of AKT. Furthermore, PGAM1 suppression can reshape the immune landscape of HCC in a ferroptosis‐related manner and significantly improve the anti‐PD‐1 immunotherapeutic efficacy in HCC, which provides preclinical evidence for the application of this combination therapy.

## 2 Results

### 2.1 PGAM1 is a Novel Immunometabolic Target Correlated with a Poor Prognosis in HCC

**Figure 1:**
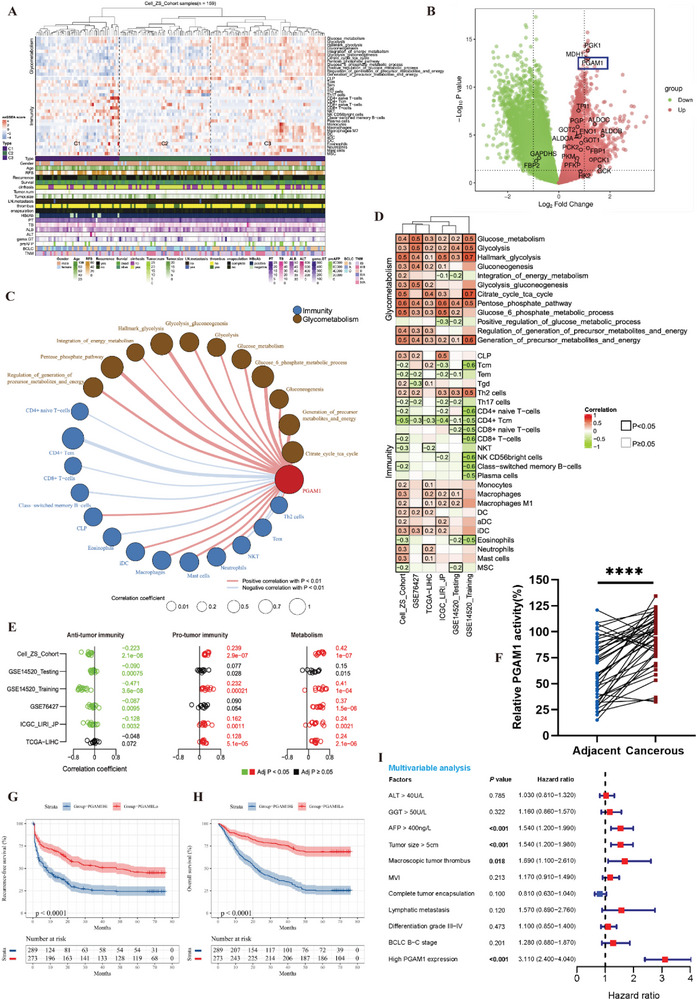
PGAM1 is a novel immunometabolic target correlated with a poor prognosis in HCC. A) Hierarchical clustering heatmap of characteristics of immune infiltration, glucose, and energy metabolism identified by ssGSEA and xCell algorithm for three clusters. C1, “low glucometabolism with anti‐tumor immune status”; C2, intermediate cluster; C3, “high glucometabolism with pro‐tumor immune status”. B) Volcano plot of differentially expressed genes (DEGs) analysis between C1 and C3. DEGs were defined as genes with adj. *p* < 0.05 and |log2FC| ≥1 (C3 vs C1) by using “limma” package. C) Correlation analysis network of PGAM1 expression, immune cell infiltration and glucometabolism in Cell‐ZS‐Cohort (based on xCell algorithm). D,E) Correlation heatmap (D) and forest plot (E) for PGAM1 expression, anti‐tumor immunity, pro‐tumor immunity, and metabolism in five HCC datasets (based on xCell algorithm). F) Multiple enzymes coupled assay was performed to detect the activities of PGAM1 in cancerous tissue and adjacent normal tissue in patients with HCC (n = 40, *p* < 0.0001, *p* values were obtained from paired *t*‐test). G,H) KaplanMeier recurrence‐free survival (RFS) (G) and overall survival (OS) (H) for HCC patients with high and low PGAM1 expression in the Zhongshan TMA cohort. I) Multivariable Cox regression analysis for clinicopathological characteristics correlated to OS of HCC patients in the Zhongshan TMA cohort.

To decipher the immunometabolic landscape of HCC, a large‐scale transcriptomic dataset (159 HCC samples) from our liver cancer center (Cell‐ZS‐Cohort) was utilized, and HCC samples were categorized based on their immune and metabolic statuses.^[^ ^8^ ^]^ Single‐sample gene set enrichment analysis (ssGSEA)^[^ ^9^ ^]^ and the xCell^[^ ^10^ ^]^ algorithm were conducted to assess the transcriptome of HCC samples to evaluate the 24 immune cell clusters and glucometabolic characteristics. To further unveil the relationship between these immune cell subsets and glucometabolism, we implemented hierarchical clustering analysis based on the enrichment score to cluster HCC samples into three different groups (clusters 1, 2, and 3) (**Figure** **1**A). C1 was characterized as “low glucometabolism and antitumor immune status”, while C3 was characterized as “high glucometabolism and protumor immune status”. C2 was categorized as the “intermediate cluster”. Gene expression profiles were compared between C1 and C3 to identify glucometabolic genes regulating immune status in HCC. The three most differentially expressed genes were PGAM1, PGK1, and MDH1 (Figure 1B). In addition to the Cell‐ZS‐Cohort dataset, four other HCC datasets, GSE14520, GSE76427, ICGC‐RI‐JP, and TCGA‐LIHC, were used to validate the results of the correlation analysis. Until now, there has been no evidence suggesting the immunological relevance of malate dehydrogenase 1 (MDH1), and our results demonstrated a weak correlation between MDH1 and protumor immunity (Figure S1A,B, Supporting Information). Phosphoglycerate kinase 1 (PGK1) was moderately correlated with pro‐tumor and antitumor immunity (Figure S1C,D, Supporting Information), but its immunological relevance and mechanisms have been well documented in multiple cancers.^[^ ^11^ ^]^ The immunological role of PGAM1 has not been recognized until now, but we identified a close correlation between PGAM1 and immunometabolic characteristics, which drew our attention. The results of xCell algorithm correlation analysis based on the Cell‐ZS‐Cohort dataset showed that PGAM1 was potentially negatively correlated with the infiltration of CD8^+^ T‐cells, CD4^+^ T central memory (CD4^+^ Tcm) cells, natural killer T (NKT) cells, T effector memory (Tem) cells, CD4^+^ T naïve cells, class‐switched B cells and eosinophils (Figure 1C,D) and was potentially positively correlated with multiple glucometabolic pathways as well as the infiltration of macrophages (Mφ), T helper 2 (Th2) cells, neutrophils, mast cells, common lymphoid precursor (CLP) cells, and immature dendritic cells (iDCs) (Figure 1C,D). Then, we validated the close correlation between PGAM1 and immunometabolic characteristics in four other HCC datasets (Figure 1E).

We also checked the clinical relevance of PGAM1 in patients with HCC to explore the potential of targeting PGAM1 for treatment. We assessed the activities of PGAM1 in cancerous and adjacent normal tissue collected from 40 patients. PGAM1 activity in cancerous tissue samples was significantly higher than that in matched adjacent normal tissue samples (Figure 1F). Corresponding immunohistochemistry (IHC) results also showed that PGAM1 expression levels in cancerous tissue were higher than those in adjacent normal tissue (Figure S1E, Supporting Information). To further delineate the main cell type (tumor cells, lymphocytes, endothelial cells, stromal cells, etc.) expressing PGAM1 in tumor microenvironment, we analyzed the single‐cell‐seq dataset from clinical HCC samples.^[^ ^12^ ^]^ Results showed that PGAM1 was more expressed in tumor cells, cancer‐associated fibroblasts (CAFs) and T‐cells (Figures S1F and S7A,B, Supporting Information). We then performed a tissue microarray‐based IHC study of 562 HCC patients (Zhongshan cohort) to investigate the prognostic value of PGAM1 expression. Kaplan‒Meier survival analysis demonstrated that high PGAM1 expression was associated with poor 5‐year recurrence‐free survival (RFS) and overall survival (OS) (Figure 1G,H). Univariate Cox regression analysis showed that ALT, GGT, AFP, tumor size, tumor encapsulation, macroscopic tumor thrombus, microvascular invasion (MVI), lymphatic metastasis, differentiation grade, BCLC stage and PGAM1 expression were significantly correlated with the survival of HCC patients (Figure S1G and Table S1, Supporting Information). Multivariable Cox regression analysis identified PGAM1 as an independent prognostic indicator for HCC patients (Figure 1I and Table S1, Supporting Information). Additionally, high PGAM1 expression was strongly associated with tumor size, macroscopic tumor thrombus, microvascular invasion and BCLC stage (**Table** **1**). With the above evidence, we concluded that PGAM1 is a potential immunometabolic target and poor prognostic biomarker in HCC.

**Table 1:** Correlation between PGAM1 expression and clinicopathological features in HCC patients.

Characteristics	Total	PGAM1 expression		*p* value
		Low (n = 273)	High (n = 289)	
**Age [year]**				0.676
≤ 60	406 (72.2%)	195 (71.4%)	211 (73.0%)	
> 60	156 (27.8%)	78 (28.6%)	78 (27.0%)	
Gender				0.835
Male	482 (85.7%)	235 (86.1%)	247 (85.5%)	
Female	80 (14.2%)	38 (13.9%)	42 (14.5%)	
HBsAg				0.196
(+)	453 (80.6%)	214 (78.4%)	239 (82.7%)	
(‐)	109 (19.4%)	59 (21.6%)	50 (17.3%)	
HCV				0.313
(+)	11 (2.0%)	7 (2.6%)	4 (1.4%)	
(‐)	551 (98.0%)	266 (97.4%)	285 (98.6%)	
TB, µmol L^−1^				0.714
≤ 21	505 (89.9%)	244 (89.4%)	261 (90.3%)	
> 21	57 (10.1%)	29 (10.3%)	28 (9.7%)	
ALB, g L^−1^				0.705
≤ 35	71 (12.6%)	33 (12.1%)	38 (13.1%)	
> 35	491 (87.4%)	240 (87.9%)	251 (86.9%)	
ALT, U L^−1^				0.335
≤ 40	236 (42.0%)	109 (39.9%)	127 (43.9%)	
> 40	326 (58.0%)	164 (60.1%)	162 (56.1%)	
GGT, U L^−1^				0.083
≤ 50	392 (69.8%)	181 (66.3%)	211 (73.0%)	
> 50	170 (30.2%)	92 (33.7%)	78 (27.0%)	
PT, s				0.691
≤ 14	514 (91.5%)	251 (91.9%)	263 (91.0%)	
> 14	48 (8.5%)	22 (8.1%)	26 (9.0%)	
AFP, ng mL^−1^				0.529
≤ 400	384 (68.3%)	190 (69.6%)	194 (67.1%)	
> 400	178 (31.7%)	83 (30.4%)	95 (32.9%)	
Cirrhosis				0.272
Yes	381 (67.8%)	179 (65.6%)	202 (69.9%)	
No	181 (32.2%)	94 (34.4%)	87 (30.1%)	
Tumor size, cm				0.001
≤ 5	314 (55.9%)	173 (63.4%)	141 (48.8%)	
> 5	248 (44.1%)	100 (36.6%)	148 (51.2%)	
Tumor number				0.224
Single	472 (84.0%)	224 (82.1%)	248 (85.8%)	
Multiple	90 (16.0%)	49 (17.9%)	41 (14.2%)	
Tumor encapsulation				0.055
Complete	273 (48.6%)	144 (52.7%)	129 (44.6%)	
Incomplete or None	289 (51.4%)	129 (47.3%)	160(55.4%)	
Macroscopic tumor thrombus				<0.001
Yes	87 (15.5%)	25 (9.2%)	62 (21.5%)	
No	475 (84.5%)	248 (90.8%)	227 (78.5%)	
MVI				0.022
Yes	204 (36.3%)	86 (31.5%)	118 (40.8%)	
No	358 (63.7%)	187 (68.5%)	171 (59.2%)	
Lymph node metastasis				0.298
Yes	19 (3.4%)	7 (2.6%)	12 (4.2%)	
No	543 (9.7%)	266 (97.4%)	277 (95.8%)	
Differentiation grade				0.471
III	408 (76.2%)	202 (74.0%)	206 (71.3%)	
IIIIV	154 (27.4%)	71 (26.0%)	83 (28.7%)	
BCLC stage				0.012
0+A	409 (72.8%)	212 (77.7%)	197 (68.2%)	
B+C	153 (27.2%)	61 (22.3%)	92 (31.8%)	

### 2.2 Targeting PGAM1 Inhibits HCC Progression and Favors the Antitumor Immune Response by Reshaping the Immune Microenvironment in HCC

**Figure 2:**
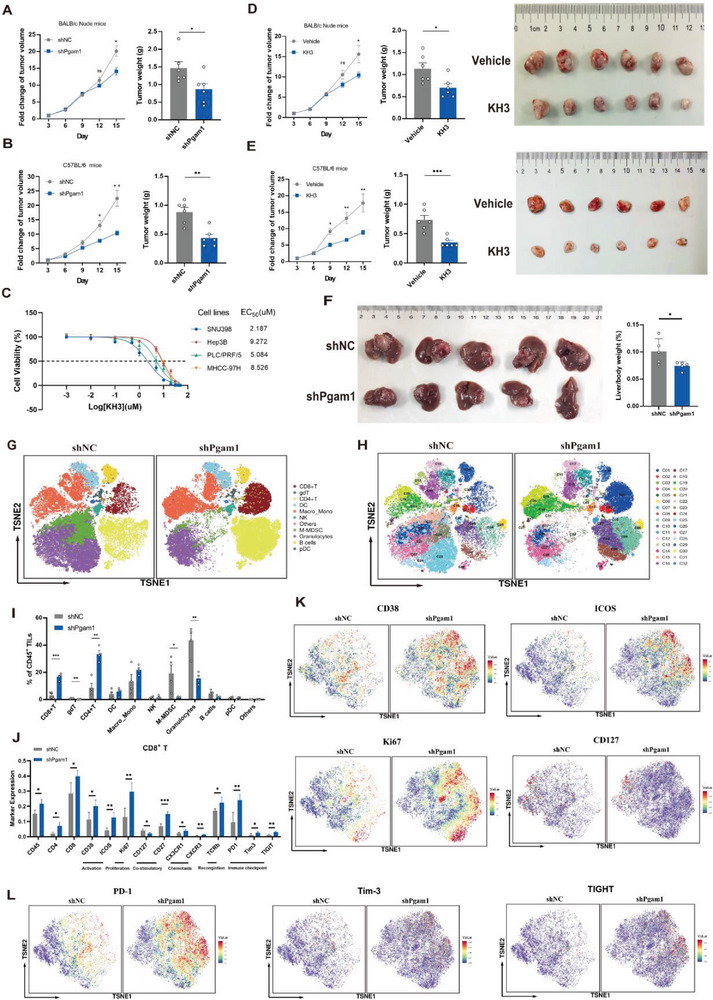
Targeting PGAM1 inhibits HCC progression and favors the antitumor immune response by reshaping the immune microenvironment in HCC. A,B) Tumor growth curve and tumor weight in immunodeficient nude mice (A) or immunocompetent C57BL/6 mice (B) with subcutaneous inoculation of shNC or shPgam1 Hepa16 cells (n = 6 per group). C) EC_50_ curve of KH3 suppressing the proliferation of 4 human HCC cell lines. D,E) Tumor growth curve, tumor weight, and tumor image in immunodeficient nude mice (D) or immunocompetent C57BL/6 mice (E) with subcutaneous inoculation of Hepa16 cells treated with vehicle (PLGA) or KH3 (n = 6 per group). F) Final images, liver weight, and liver/body weight (%) of C57BL/6 mice bearing orthotopic shNC and shPgam1 Hepa16 tumors. G,H) The *t*‐SNE plot of major immune cell types (G) and 32 immune subsets (H) within CD45^+^ tumor‐infiltrating leukocytes of shNC or shPgam1 orthotopic Hepa16 tumors by mass cytometry (CyTOF) (n = 5 per group). I) Histogram for the frequencies of major immune cell types within the CD45^+^ population of shNC and shPgam1 Hepa16 orthotopic tumors. J) Histogram for the expression of significantly altered markers of the CD8^+^ T population between shNC and shPgam1 Hepa16 orthotopic tumors. K) Density *t*‐SNE plot for the expression of activation markers (CD38 and ICOS), proliferation markers (Ki67), and co‐stimulatory markers (CD127) in infiltrated CD8^+^ T‐cell within the CD45^+^ population of shNC and shPgam1 Hepa16 orthotopic tumors by CyTOF analysis. L) Density *t*‐SNE plot for the expression of immune checkpoints (PD‐1, Tim‐3, and TIGHT) in infiltrated CD8^+^ T‐cell within the CD45^+^ population of shNC and shPgam1 Hepa16 orthotopic tumors by CyTOF analysis. The data were presented as the means ± SD and *p* values were determined by a two‐tailed unpaired *t*‐test.

To investigate the immune relevance of PGAM1, we targeted Pgam1 (both genetically and pharmacologically) to observe tumor growth and tumor weight in a xenograft experiment in which C57BL/6 mice were subcutaneously injected with Hepa16 cells. Genetic knockdown of PGAM1 was performed by stable transfection of Hepa16 cells with a vector encoding PGAM1 short hairpin RNA (shPgam1) (Figure S2A, Supporting Information). The growth of the tumor mass in nude or C57BL/6 mice was monitored after subcutaneous inoculation of Hepa16 cells for 1 week. We identified that genetic knockdown of Pgam1 significantly decreased tumor growth in C57BL/6 mice (immunocompetent) compared with that in nude (immunodeficient) mice (**Figure** **2**A,B; Figure S2B,C, Supporting Information). In parallel, PLC/PRF/5 cells stably transfected with shPGAM1 were subcutaneously injected into nude mice, and the findings were consistent with previous findings (Figure S2D, Supporting Information). Similar to genetic knockdown of Pgam1, pharmacological inhibition of Pgam1 also inhibited HCC growth in vivo. Six days after inoculation, KH3, a novel allosteric PGAM1 inhibitor established by our previous studies,^[^ ^7^ ^]^ was intraperitoneally administered at a dose of 75 mg kg^−1^ once every 3 days 3 times (Figure S2E, Supporting Information). To determine the efficacy of PGAM1 inhibitors on cell proliferation, we first treated 7 human HCC cell lines and 2 murine HCC cell lines with KH3. The results showed that KH3 effectively suppressed human HCC cell proliferation with EC_50_ values ranging from 2.187 to 9.272 µm (Figure 2C; Figure S2F, Supporting Information). Next, we tested the ability of KH3 to inhibit primary HCC cells isolated from six patients. Likewise, the primary HCC cells were more sensitive to KH3, with EC_50_ values ranging from 1.322 to 3.896 µm (Figure S2G, Supporting Information), than the commercial cancer cells. As expected, KH3 treatment suppressed HCC tumor growth (Figure 2D,E) and significantly prolonged survival to a greater extent in C57BL/6 mice than in nude mice (Figure S2H, Supporting Information). We also administered KH3 to C57BL/6 mice orthotopically injected with Hepa16 cells, which verified the antitumor effect of KH3 (Figure S2I, Supporting Information). No significant difference in body weight was observed in tumor‐bearing mice with genetic or pharmacological inhibition of PGAM1 (Figure S2J, Supporting Information). There was no difference between the KH3 treatment group and vehicle group in terms of the serum levels of ALT, AST and creatinine, indicating the good safety of KH3 (Figure S2K, Supporting Information). These results suggest that both genetic knockdown and pharmacological suppression of PGAM1 contribute to the inhibition of tumor growth, in which the potential participation of immune mechanisms might be involved. To investigate the underlying functions of PGAM1 in the liver microenvironment, we first evaluated the landscape of immunological alterations between orthotopic tumors derived from Pgam1 knockdown and mock knockdown Hepa16 cells. We found a significantly lower liver/body weight ratio in the group with shPgam1 orthotopic HCC tumors (Figure 2F). The orthotopic tumors were dissociated and subjected to immune cell profiling by time‐of‐flight mass cytometry (CyTOF), in which 42 selected monoclonal antibodies (mAbs) were applied to reveal 11 major immune clusters (Figure 2G) and 32 distinct immune cell subsets (Figure 2H) after PhenoGraph clustering analysis. We observed a noticeable increase in CD8^+^ T and CD4^+^ T‐cells and a decrease in MDSCs and granulocytes in shPgam1 tumors (Figure 2I). Comprehensive analysis of the whole CD45^+^ immune cell population allowed a detailed comparison of a total of 32 immune cell subsets, such as CD38^lo^Ly6C^+^Ki67^+^PD1^+^CD8^+^ T‐cells (C1 cluster) (Figure S2L,M and Table S2, Supporting Information). Furthermore, we found that the alteration of CD8^+^ T‐cell infiltration was the most significant among these immune cells (Figure 2I), so we further analyzed the significant alteration of marker expression in CD8^+^ T‐cells (Figure 2J). Compared with those of the control group, the infiltrated CD8^+^ T‐cells of shPgam1 tumors had higher expression of activation markers (CD38 and ICOS), proliferation markers (Ki67), functional recognition marker (TCRb) and costimulatory markers (CD127 and CD27) (Figure 2K; Figure S2N, Supporting Information). Additionally, we observed higher expression of chemokine receptors (CXCR3 and CX3CR1) (Figure S2O, Supporting Information) and immune checkpoints (PD‐1, Tim‐3, and TIGHT) in infiltrated CD8^+^ T‐cells of shPgam1 tumors (Figure 2L). Together, these results suggest that PGAM1 inhibition facilitates reconstruction of the immune microenvironment of HCC.

### 2.3 PGAM1 Inhibition Enhances Ferroptosis in HCC Cells

**Figure 3:**
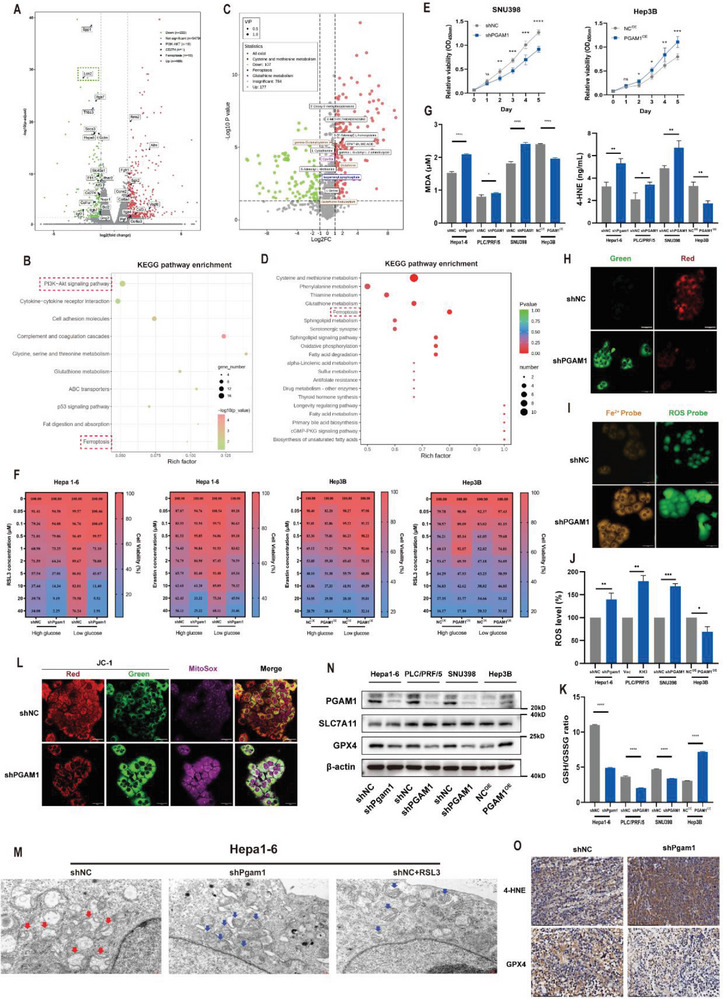
PGAM1 inhibition enhances ferroptosis in HCC cells. A) Volcano plot of RNA data for shNC and shPgam1 Hepa16 cells. A total of 691 significant DEGs was detected by using “DESeq2” package, including 469 upregulated genes and 222 downregulated genes. B) KEGG analysis for the identification of PGAM1‐mediated downstream pathways based on the DEG analysis result of Hepa16 cells. C) Volcano plot of metabonomic data for shNC and shPgam1 Hepa16 cells. D) KEGG analysis for the identification of PGAM1‐mediated downstream pathways based on the differential metabolites analysis result of Hepa16 cells. E) Cell viability changes (CCK‐8 assays) of PGAM1‐silencing SNU398 and PGAM1 overexpressed Hep3B cell line over 4 days. F) Heatmap demonstrated targeting PGAM1 enhanced the sensitivity to two ferroptosis inducers (RSL3, Erastin) in Hepa16 and Hep3B cell lines under high glucose and low glucose media. G) The concentration of MDA and 4‐HNE were detected in PGAM1‐silencing Hepa16, PLC/PRF/5, SNU398 cell lines, and PGAM1 overexpressed Hep3B cell line. The protein concentration of each cellular lysis was titrated to 500ug ul^−1^. H) BODIPY 581/591 C11 (a marker of lipid peroxidation) fluorescent probe showing increased lipid peroxidation accumulation in PLC/PRF/5 cells with shPGAM1 (Scale bar = 50 µm). I) FerroOrange and ROS probe exhibiting increased intracellular Fe^2+^ levels and ROS levels in PLC/PRF/5 cells with shPGAM1 (Scale bar = 50 µm). J,K) Quantitation of ROS levels and GSG/GSSG ratio were detected in PGAM1‐silencing Hepa1‐6, PLC/PRF/5, SNU398 cell lines, and PGAM1 overexpressed Hep3B cell line. The protein concentration of each cellular lysis was titrated to 500ug ul^−1^. L) JC‐1 detections with mitoSOX staining comprehensively showing ROS accumulation and reduced membrane potential in mitochondria in PLC/PRF/5 cells with shPGAM1 (Scale bar = 50 µm). M) TEM imaging was conducted in shNC and shPgam1 Hepa16 cells. Hepa16 cells treated with RSL3 (2 µmol L^−1^) functioned as the positive control. Red arrows showed the morphology of normal mitochondria in shNC Hepa16 cells. Blue arrows showed the shrunk mitochondria in shPgam1 Hepa16 cells and positive control (Scale bar = 2 µm). N) Western blot showing the expression of SLC7A11 and GPX4 in the indicated four modified HCC cell lines. O) IHC examining the level of 4‐HNE and the expression of GPX4 in mouse subcutaneous tumor tissues of Hepa16 cells with shNC or shPgam1 (Scale bar = 100 µm). The data were presented as the means ± SD of three independent experiments or triplicates. *p* values were determined by a two‐tailed unpaired *t*‐test. ^*^ *p* < 0.05; ^**^ *p* < 0.01; ^***^ *p* < 0.001; ^****^ *p* < 0.0001; n.s., not significant, *p* > 0.05.

PGAM1 was initially considered a critical metabolic enzyme involved in glycolysis and biosynthesis, but its inhibition has been gradually acknowledged to cosuppress several metabolic and cancerous pathways.^[^ ^6^ ^]^ To illustrate the potential mechanism by which PGAM1‐mediated metabolic remodeling contributes to HCC progression, we performed transcriptomic and untargeted metabolomic analysis of shNC and shPgam1 Hepa16 subcutaneous tumors in C57BL/6 mice (n = 3 per group). KEGG enrichment analysis of both differentially expressed genes (DEGs) and metabolites implied that the ferroptosis pathway and T‐cell proliferation were preferentially activated in PGAM1‐knockdown cells (**Figure** **3**A–D; Figure S3A,B, Supporting Information). The protein and mRNA levels of PGAM1 were significantly increased in several human HCC cell lines compared to L‐02 normal liver cells (Figure S3C, Supporting Information). To examine the role of PGAM1 in HCC in vitro, four HCC cell lines, Hepa16, PLC/PRF/5, SNU398 and Hep3B cells, were used to validate the functional phenotype. First, we confirmed that knockdown of PGAM1 in the PLC/PRF/5 and SNU398 human HCC cell lines and Hepa1‐6 mouse HCC cell lines significantly decreased cell proliferation, while overexpression of PGAM1 in the Hep3B human HCC cell line significantly increased cell proliferation (Figure 3E; Figure S3D, Supporting Information). To verify whether this impact on proliferation is correlated with PGAM1‐mediated ferroptosis, we tested the killing effect of two common ferroptosis inducers (RSL3 and erastin) in four HCC cell lines under high‐ and low‐glucose conditions, as a previous study indicated that tumor cells could exhibit ferroptosis resistance under glucose starvation.^[^ ^13^ ^]^ We found that PGAM1 inhibition significantly sensitized HCC cells to two ferroptosis inducers regardless of glucose concentration. The cell viability of sh‐PGAM1 was significantly lower than that of shNC, especially when the concentration of ferroptosis inducers was high (Figure 3F; Figure S3E, Supporting Information). Next, we quantified malondialdehyde (MDA) and 4‐hydroxynonenal (4‐HNE), two of the most commonly used hallmarks of lipid peroxidation. The results of biochemical experiments illustrated that PGAM1‐knockdown HCC cells exhibited significantly increased MDA and 4‐HNE levels (Figure 3G), while overexpression of PGAM1 in Hep3B cells triggered a decrease. The BODIPY 581/591 assay showed consistent results (Figure 3H; Figure S3F, Supporting Information). In addition, PGAM1 inhibition increased intracellular ROS levels, Fe^2+^ levels, and total iron content (Figure 3I,J; Figure S3G, Supporting Information) while decreasing the intracellular GSH/GSSG ratio (Figure 3K). PGAM1^OE^ Hep3B cells showed the opposite trend (Figure 3J,K; Figure S3G,H, Supporting Information). Ferroptosis is often associated with ROS accumulation and reduced membrane potential in mitochondria,^[^ ^30^ ^]^ which was comprehensively detected by JC‐1 with mitoSOX assays (Figure 3L). As expected, PGAM1 inhibition increased ROS levels and reduced membrane potential and bioactivity in mitochondria, while overexpressing PGAM1 exhibited the opposite effects (Figure S3I,J, Supporting Information). To verify the ferroptosis phenotype morphologically, transmission electron microscopy (TEM) was conducted for shPGAM1 Hepa1‐6 cells. PGAM1 suppression led to cell shrinkage and mitochondrial shrinkage (with increased mitochondrial membrane density), and these cells resembled the positive control cells (cultured with 2 µm RSL3) (Figure 3M). Next, we tested the expression of cystine/glutamate antiporter xCT (SLC7A11) and glutathione peroxidase 4 (GPX4), both of which are key markers in the pathway associated with ferroptosis.^[^ ^13^, ^14^ ^]^ PGAM1 inhibition reduced the expression of GPX4 but did not change the level of SLC7A11 (Figure 3N; Figure S3K, Supporting Information). We also examined the expression of 4‐HNE and GPX4 in mouse subcutaneous tumor tissues, as shown in Figure 2B,E, and found that PGAM1 suppression led to higher levels of lipid peroxidation and lower expression of GPX4 (Figure 3O; Figure S3L, Supporting Information).

### 2.4 Targeting PGAM1 Suppresses LCN2 Expression via Energy Stress/ROS‐Dependent Inhibition of AKT

**Figure 4:**
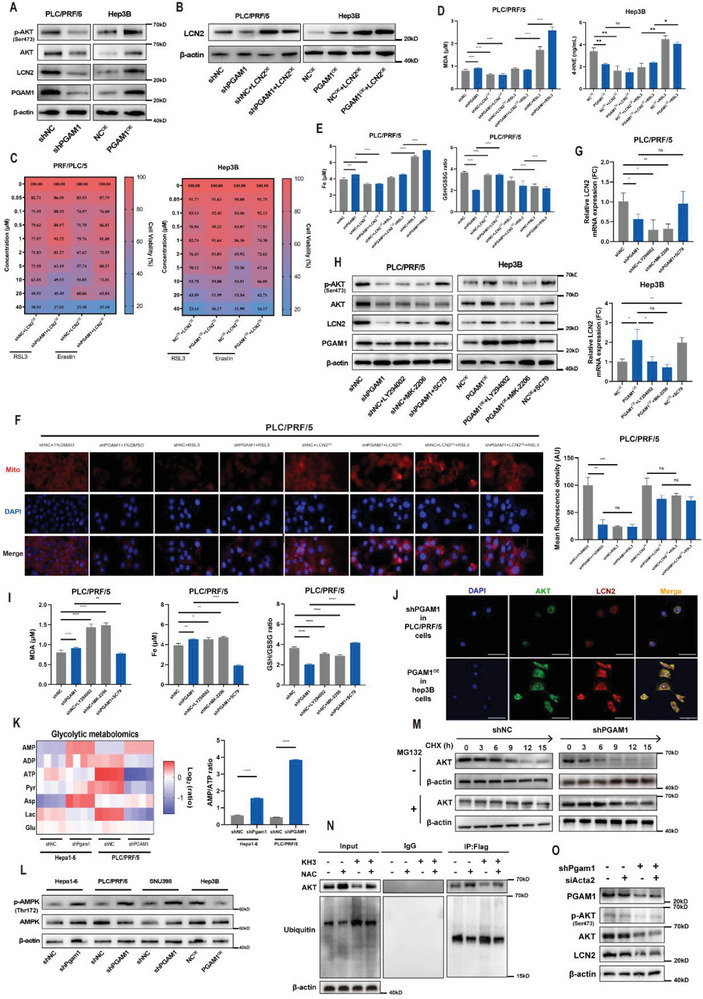
Targeting PGAM1 suppresses LCN2 expression via energy stress/ROS‐dependent inhibition of AKT. A) Western blot detected the protein expression of PGAM1, LCN2, AKT, p‐AKT in indicated PLC/PRF/5 and Hep3B cell lines. B) Western blot validated the establishment of the above 2 HCC cell lines with recombinant LCN2 expression. C) Heatmap demonstrated recombinant LCN2 expression reversed the enhancement of ferroptosis sensitivity induced by PGAM1 inhibition in the above 2 HCC cell lines under high glucose condition. D) The intracellular concentration of MDA and 4‐HNE were detected in indicated PLC/PRF/5 or Hep3B cell lines treated with RSL3 (2 µm) in the presence or absence of recombinant LCN2 expression. The protein concentration of each cellular lysis was titrated to 500ug ul^−1^. E) Total iron and GSH/GSSG ratio were detected in indicated PLC/PRF/5 treated with RSL3 (2 µm) in the presence or absence of recombinant LCN2 expression. The protein concentration of each cellular lysis was titrated to 500ug ul^−1^. F) Mitochondrial membrane potential detection and statistics in indicated PLC/PRF/5 cells treated with RSL3 (2 µm) in the presence or absence of recombinant LCN2 expression (Scale bar = 50 µm). G) Relative mRNA expression of LCN2 in the above 2 HCC cell lines treated with LY294002 (15 µm), MK‐2206 (15 µm), or SC79 (15 µm). H) Western blot detected the protein expression of PGAM1, LCN2, AKT, p‐AKT in indicated PLC/PRF/5 and Hep3B cell lines treated with LY294002 (15 µm), MK‐2206 (15 µm) or SC79 (15 µm). I) The intracellular concentration of MDA, total iron, and GSH/GSSG ratio was detected in indicated PLC/PRF/5 cell lines treated with LY294002 (15 µm), MK‐2206 (15 µm) or SC79 (15 µm). J) IF staining exhibiting the expression characteristics and co‐localization of AKT and LCN2 in PGAM1 differentially expressed PLC/PRF/5 and Hep3B cells (Scale bar = 50 µm). K) Metabolic heatmap for several glycolytic intermediates and the AMP/ATP ratio in indicated Hepa16 and PLC/PRF/5 cell lines (n = 3 per group). L) Western blot detected the protein expression of AMPK and p‐AMPK in indicated Hepa16, PLC/PRF/5, SNU398, and Hep3B cell lines. M) Western blotting showing the effect of PGAM1 depletion on AKT stability in PLC/PRF/5 cells incubated with CHX or MG132 at the indicated time points. N) Co‐IP analyses were conducted to identify the function of KH3 (pharmacological PGAM1 inhibition) and NAC (ROS scavenger) on the AKT ubiquitination level in PLC/PRF/5 cells incubated with MG132. O) Western blotting showing the effect of PGAM1 depletion and/or ACTA2 depletion on the expression of p‐AKT, AKT, and LCN2 in PLC/PRF/5 cells. The data were presented as the means ± SD of three independent experiments or triplicates. *p* values were determined by a two‐tailed unpaired *t*‐test. ^*^ *p* < 0.05; ^**^ *p* < 0.01; ^***^ *p* < 0.001; ^****^ *p* < 0.0001; n.s., not significant, *p* > 0.05.

Among the DEGs involved in the ferroptosis pathway, LCN2, an iron‐sequestering cytokine rendering insensitivity to ferroptosis by depleting iron, was significantly downregulated among all ferroptosis‐related genes (Figure 3A). KEGG analysis revealed that the most significantly enriched pathway was the PI3K‐AKT signaling pathway (Figure 3B), which has been reported to regulate LCN2 expression.^[^ ^13^ ^]^ We also validated a potential correlation of PGAM1 with LCN2, AKT, and ferroptosis markers based on five HCC datasets (Figure S8, Supporting Information). Decreased protein expression of AKT, p‐AKT, and LCN2 was observed in three PGAM1‐silenced HCC cell lines, while elevated expression was detected in PGAM1‐overexpressing Hep3B cells (**Figure** **4**A; Figure S4A, Supporting Information). Based on the evidence above, we speculated that PGAM1 inhibition may reverse LCN2‐mediated ferroptosis resistance by downregulating AKT.

Considering the above findings, we established four HCC cell lines with recombinant LCN2 expression (Figure 4B; Figure S4B, Supporting Information), and elevated LCN2 expression rescued resistance to RSL3 and erastin, especially in shPGAM1 HCC cells (Figure 4C; Figure S4C, Supporting Information). We utilized HCC cells cultured with RSL3 as a positive control for ferroptosis and found that LCN2 overexpression led to a significant decrease in the intracellular levels of MDA, 4‐HNE (Figure 4D; Figure S4D, Supporting Information) and total iron (Figure 4E; Figure S4E, Supporting Information), as well as an increase in the GSH/GSSG ratio (Figure S4F, Supporting Information). LCN2 overexpression also enhanced ferroptosis resistance to RSL3 (Figure 4D,E; Figure S4D–F, Supporting Information). In addition, LCN2 overexpression reversed the decrease in mitochondrial membrane potential induced by PGAM1 inhibition and RSL3 stimulation in Hepa16 and PLC/PRF/5 cell lines (Figure 4F; Figure S4G, Supporting Information).

Next, we identified that LCN2 mRNA and protein expression was decreased following the administration of two AKT inhibitors (LY294002 and MK‐2206) in shNC HCC cells and was upregulated following the administration of an AKT activator (SC79) (Figure 4G,H; Figure S4H,I, Supporting Information). As expected, AKT inhibitors significantly increased the intracellular levels of MDA and iron and reduced the GSH/GSSG ratio (Figure 4I; Figure S4J,L, Supporting Information), while AKT activators reversed the alteration of these ferroptosis indicators in shPGAM1 HCC cells. The immunofluorescence (IF) results also showed the expression signature and colocalization of AKT and LCN2 in HCC cells with altered PGAM1 expression (Figure 4J). Furthermore, we explored the potential mechanism by which targeting PGAM1 regulates AKT. We applied mass spectrometry of glycolytic metabolism‐related factors to confirm the glucose and energy alterations after PGAM1 knockdown in Hepa16 and PLC/PRF/5 cells (Figure 4K). PGAM1 inhibition led to significant energy stress and metabolic alterations, manifesting as increased levels of adenosine monophosphate (AMP) and aspartate and decreased levels of adenosine triphosphate (ATP). It is well known that an increase in the AMP/ATP ratio (Figure 4K) and accumulation of aspartate can trigger the activation of AMPK,^[^ ^15^ ^]^ which is recognized as an energy sensor that exerts an antagonistic effect to regulate AKT activity under metabolic stress. We subsequently verified the increase in AMPK and phospho‐AMPK (p‐AMPK) levels, which supported the mechanism of energy stress (Figure 4L).

We speculated that PGAM1 inhibition affects the gene transcription and/or protein stability of AKT, thereby decreasing AKT protein levels. First, we determined the effect of genetic and pharmacological PGAM1 inhibition on the mRNA expression of three AKT isoforms (AKT13) in PLC/PRF/5 and Hepa16 cells by qRT‒PCR. The results showed that suppressing PGAM1 had little effect on the transcription of AKT (Figure S4M, Supporting Information), indicating that PGAM1 inhibition modulated AKT expression at the posttranscriptional level. Next, we employed a cycloheximide (CHX) chasing assay on PLC/PRF/5 and Hepa16 cells. shPGAM1 or KH3 treatment accelerated the degradation of AKT (Figure 4M; Figure S4N, Supporting Information). This indicated that inhibiting PGAM1 promoted AKT proteolysis via the ubiquitin‒proteasome degradation pathway, which was reported in a previous study.^[^ ^16^ ^]^ Previous research confirmed that short‐term treatment with HKB99 (another allosteric inhibitor of PGAM1) at 5 mm for 6 h significantly increased the ROS level and decreased the p‐AKT level,^[^ ^6^ ^]^ and the effect was diminished by combination with the ROS scavenger N‐acetyl‐l‐cysteine (NAC). The above findings are consistent with our results (Figures S4O and S7C, Supporting Information). Thus, we performed Co‐IP and Western blotting to examine the effect of KH3 on AKT ubiquitination, which showed that the AKT ubiquitination level was elevated upon KH3 treatment, and this effect could be reversed by NAC treatment (Figure 4N).

A previous report indicated that PGAM1 promotes cell migration independent of its metabolic activity mainly through interaction of residues 201–210 with ACTA2 in breast cancer cells.^[^ ^10^ ^]^ We aimed to distinguish whether this decrease in AKT expression is mediated by canonical metabolic activity or noncanonical PGAM1 activity related to its interaction with ACTA2. Three independent siRNAs were used to silence ACTA2 (Figure S4P, Supporting Information). Co‐IP and Western blots showed that decreased AKT expression by PGAM1 inhibition is mainly mediated by the decrease in enzymatic activity (Figure 4O; Figures S4Q and S7D, Supporting Information). In order to further confirm that previous phenotypes of ferroptosis were associated with the canonical metabolic activity of PGAM1 rather than its interaction with ACTA2, catalytically inactive H186R and Δ201–220 mutants lacking ACTA2 binding ability were individually transfected into cells with stable knockdown of PGAM1 (Figure S7E, Supporting Information). We tested several ferroptotic phenotypes and the results were consistent with our assumptions (Figure S7F–K, Supporting Information).

### 2.5 PGAM1 Inhibition could Promote CD8^+^ T‐Cell Infiltration and Downregulate PD‐L1 in HCC

**Figure 5:**
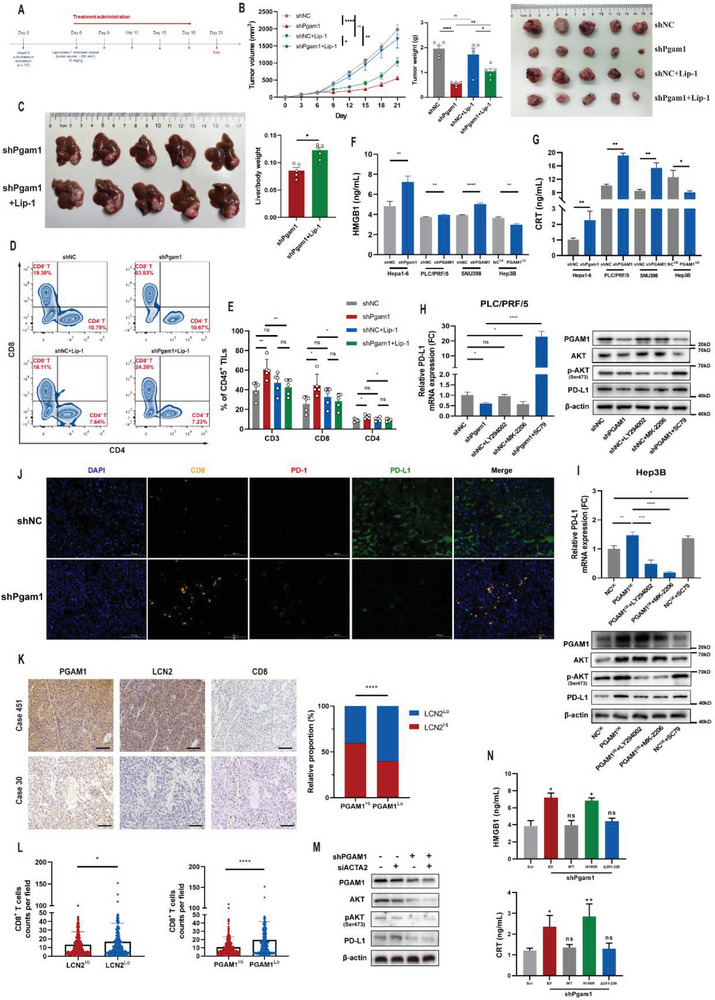
PGAM1 inhibition could promote CD8^+^ T‐cell infiltration and down‐regulate PD‐L1 in HCC. A) Liproxstatin‐1 treatment strategy of HCC growth inhibition in subcutaneous shNC and shPgam1 Hepa16 xenografts (n = 5 per group). Six days after shNC and shPgam1 Hepa16 cell inoculation, liproxstatin‐1 was intraperitoneally injected (15 mg kg^−1^) once every 3 days for 5 times. Mice were sacrificed on day 21. B) Tumor growth curves, tumor weight and tumor images of Hepa16 subcutaneous xenografts of ferroptosis inhibition experiment (n = 5 per group). C) Final images and liver/body weight (%) of C57BL/6 mice with orthotopic injection of shPgam1 Hepa16 cells and treated with Liproxstatin‐1 (n = 5 per group). D,E) Representative images and quantification of CD8^+^ tumor‐infiltrating leukocytes analyzed by flow cytometry for subcutaneous shNC and shPgam1 Hepa16 tumors treated with liproxstatin‐1 (n = 5 per group). F,G) Cellular supernatant of HMGB1 and CRT in four PGAM1‐modified HCC cell lines. H,I) Relative mRNA of PD‐L1 as well as protein expression of PGAM1, AKT, p‐AKT and PD‐L1 in indicated PLC/PRF/5 and Hep3B cells treated with LY294002 (15 µm), MK‐2206 (15 µm) or SC79 (15 µm); the mRNA expression of the other 4 groups were normalized according to the result of shNC. J) mIHC staining exhibiting the infiltration of CD8^+^ T‐cells, PD‐1and PD‐L1 expression of Hepa16 subcutaneous tumor in shNC and shPgam1 group (Scale bar = 100 µm). K) Representative IHC images of PGAM1, LCN2, CD8 expression and the relative proportion of LCN2^Hi^ and LCN2^Lo^ HCC tissues in PGAM1^Hi^ and PGAM1^Lo^ HCC tissues of Zhongshan TMA cohort (Scale bar = 200 µm). L) The CD8^+^ T‐cell counts per field (CD8^+^ T‐cell infiltration) is negatively associated with PGAM1 and LCN2 expression in HCC tissues of Zhongshan TMA cohort. M) Western blotting showing the effect of PGAM1 depletion and/or ACTA2 depletion on the expression of AKT, p‐AKT, and PD‐L1 in PLC/PRF/5 cells. N) Cellular supernatant of HMGB1 and CRT in Pgam1 stably depleted Hepa16 cells reconstituted with PGAM1 wildtype or indicated mutants. The data were presented as the means ± SD of three independent experiments or triplicates. *p* values were determined by a two‐tailed unpaired *t*‐test, ^*^ *p* < 0.05; ^**^ *p* < 0.01; ^***^ *p* < 0.001; ^****^ *p* < 0.0001; n.s., not significant, *p* > 0.05.

Previously, we discovered that the alteration of CD8^+^ T‐cell infiltration was the most significant in the shPGAM1 group (Figure 2I). To validate whether PGAM1‐mediated CD8^+^ T‐cell infiltration is ferroptosis dependent, we detected HCC tumor growth in vivo in the absence or presence of liproxstatin‐1 (**Figure** **5**A). Inhibiting ferroptosis by liproxstatin‐1 significantly alleviated the antitumor effect of PGAM1 inhibition (Figure 5B,C; Figure S5A, Supporting Information). Flow cytometry analysis was performed to identify alterations in immune infiltration following ferroptosis suppression. We found that the promotive effect of PGAM1 inhibition on T‐cell infiltration was reversed when ferroptosis was inhibited by liproxstatin‐1 (Figure 5D,E; Figure S5B, Supporting Information).

It is generally acknowledged that ferroptotic tumor cells are capable of releasing some “find‐me” and “eat‐me” immunostimulating signals, especially damage‐associated molecular patterns (DAMPs), which recruit dendritic cells, macrophages, CD8^+^ T‐cells and other immune cells to the ferroptotic site. In the above four modified HCC cell lines, we detected the release of high‐mobility group box 1 (HMGB1) and calreticulin (CRT), two typical DAMPs involved in oxidative stress and the cell death response, which implied a ferroptosis‐mediated inflammatory response (Figure 5F,G). AKT activation or LCN2 overexpression reversed the increase in HMGB1 secretion mediated by PGAM1 knockdown in vitro (Figure S5C,D, Supporting Information).

Furthermore, we noticed higher expression of immune checkpoints on CD8^+^ T‐cells (Figure 2J–L), which inspired us to investigate the potential alteration of PD‐L1 expression on HCC cells as well. Since it has been reported that PD‐L1 is regulated by AKT^[^ ^17^ ^]^ and that abnormal PI3K/AKT pathway activation results in increased PD‐L1 expression in various tumors,^[^ ^18^ ^]^ PD‐L1 downregulation may be another potential reason for the enhancement of antitumor immunity. Indeed, we found that the mRNA and protein expression of PD‐L1 was reduced following PGAM1 or AKT inhibition, and expression could be restored by PGAM1 overexpression or AKT activation (Figure 5H,I; Figure S5E,F, Supporting Information). In addition, mIHC was performed to further confirm the infiltration of CD8^+^ T‐cells and PD‐1 and PD‐L1 expression in Hepa1‐6 subcutaneous tumors in the shNC and shPgam1 groups, and the results were consistent with our previous results (Figure 5J). Then, we assessed the correlations among PGAM1 expression and LCN2 expression and CD8^+^ T‐cell infiltration based on IHC of samples from HCC patients in the Zhongshan TMA cohort. Our results showed that PGAM1 expression was positively correlated with LCN2 expression (Figure 5K). CD8^+^ T‐cell infiltration was negatively correlated with both PGAM1 expression and LCN2 expression (Figure 5L). Moreover, we found that high LCN2 expression and low CD8^+^ T‐cell infiltration were correlated with a poor prognosis (Figure S5G,H, Supporting Information). Again, the observed phenotypes of reshaping the immune microenvironment were due to the decreased enzymatic activity of PGAM1, not interference with its association with ACTA2 (Figure 5M,N; Figure S5I, Supporting Information).

### 2.6 KH3 Exhibits Significant Antitumor effects in PDX Models and Synergizes with PD‐1 Blockade Immunotherapy

**Figure 6:**
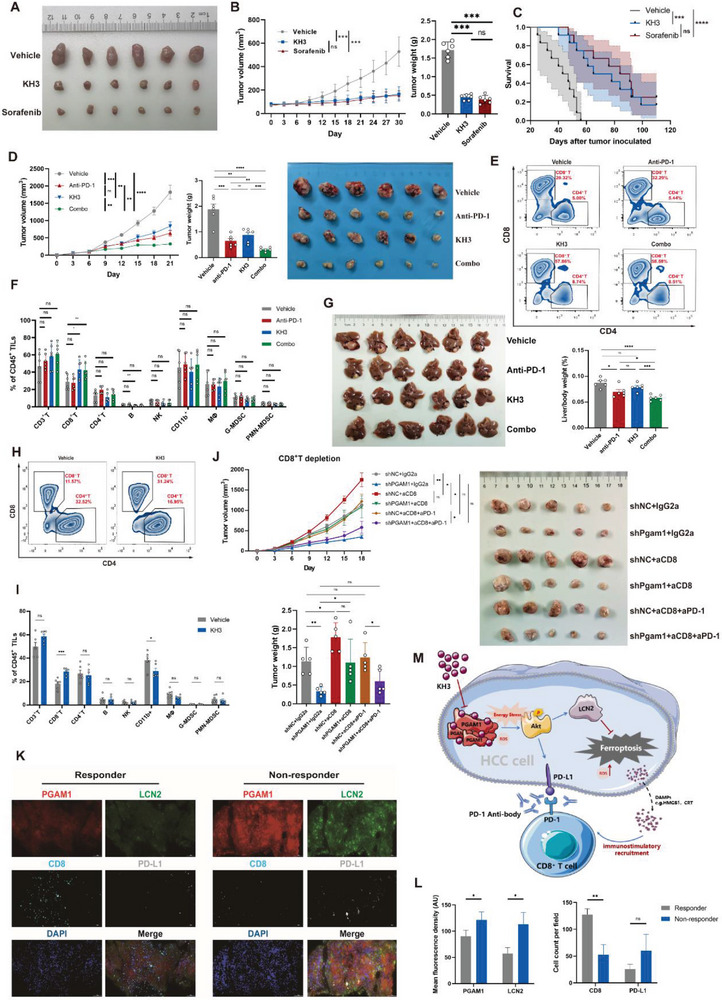
KH3 exhibits significant antitumor effects in PDX models and synergizes with PD‐1 blockade immunotherapy. A) Endpoint tumor images of the PDX models treated with vehicle (PLGA), KH3, or Sorafenib (n = 6 per group). B) Tumor growth curves and tumor weight of PDX models mentioned above (n = 6 per group). C) Kaplan−Meier curves of overall survival of PDX models mentioned above (n = 12 per group). D) Tumor growth curves, tumor weight, and tumor images of subcutaneous Hepa16 tumors treated with vehicle (PLGA) or KH3 combined with IgG2a or anti‐PD‐1 mAb (n = 6 per group). E,F) Representative images and quantification of CD45^+^ tumor‐infiltrating leukocytes analyzed by flow cytometry for subcutaneous Hepa16 tumors. G) Image and liver/body weight (%) of orthotopic Hepa16 tumors treated with vehicle (PLGA) or KH3 combined with IgG2a or anti‐PD‐1 mAb (n = 6 per group). H,I) Representative images and quantification of CD8^+^ tumor‐infiltrating leukocytes analyzed by flow cytometry for orthotopic Hepa16 tumors (n = 6 per group). J) Tumor growth curve, tumor weight, and tumor image of shNC and shPgam1 subcutaneous Hepa16 xenografts treated with anti‐CD8 mAb combined with IgG2a or anti‐PD‐1 mAb (n = 5 per group). K,L) Representative mIF images (K) and statistics (L) of PGAM1, LCN2, CD8, and PD‐L1 expression in pre‐immunotherapy punctured HCC samples of responders (n = 3) and non‐responders (n = 3). M) Schematic illustration of the PGAM1‐mediated model. Data in (B,D,F,G,I,J) are presented as the mean ± SD; data in (L) are presented as the mean ± SEM; *p* values were determined by a two‐tailed unpaired *t*‐test in (F,I); *p* values were determined by one‐way ANOVA in (B); *p* values were determined by two‐way ANOVA in Figure D, G; *p* values were determined by multi‐factor ANOVA in (J); ^*^ *p* < 0.05; ^**^ *p* < 0.01; ^***^ *p* < 0.001; ^****^ *p* < 0.0001; n.s., not significant, *p* > 0.05.

To better estimate the clinical efficacy of KH3 in the treatment of patients with HCC, we established 6 patient‐derived xenografts (PDXs) in NSG (nonobese diabetic; severe combined immunodeficiency; interleukin‐2 receptor gamma chain null) mice. The PGAM1 expression level of tumor tissue and adjacent normal tissue was assessed (Figure S6A, Supporting Information). Corresponding primary cell lines were expanded, and the EC50 of KH3 was identified (Figure S2F, Supporting Information). Then, we tested the antitumor effects of KH3 compared with vehicle and sorafenib, which is one of the first‐line therapies in HCC^[^ ^4^, ^19^ ^]^ and a ferroptosis inducer.^[^ ^20^ ^]^ In preclinical testing, we implanted the PDX tumor tissues mentioned above into NSG mice and started the treatment when the tumors reached 50–100 mm^3^. The mice were assigned to three treatment groups: 1) vehicle; 2) KH3+vehicle; and 3) sorafenib+vehicle. KH3 was intraperitoneally administered at a dose of 75 mg kg^−1^ once every 3 days until the endpoint. Sorafenib was administered at 30 mg kg^−1^ by oral gavage once a day until the endpoint. Compared with the vehicle group, KH3 and sorafenib exhibited excellent antitumor effects (**Figure** **6**A,B) and significantly prolonged survival (Figure 6C). The body weights of the above three groups did not differ until late in the experiment. Approaching the endpoint, weight loss due to cachexia was observed in the vehicle group (Figure S6B, Supporting Information). Massive necrosis was observed based on HE is staining of tumors in the KH3 and sorafenib groups compared to the vehicle group, which reconfirmed the antitumor effects of KH3 (Figure S6C, Supporting Information). Ferroptosis is not the sole mechanism of action of sorafenib. We added a liproxstatin‐1 group to demonstrate that the additive antitumor effects were mediated by enhanced ferroptosis, and the expression of 4‐HNE in tumors was detected (Figure S6D, Supporting Information). Complete blood count (CBC) and comprehensive metabolic panel (CMP) analyses showed no significant difference in the hematopoietic and biochemical properties among the three groups of mice, which demonstrated the good safety of KH3 (Table S3, Supporting Information).

The aforementioned in vivo experiments and CyTOF results indicated that PGAM1 inhibition improved antitumor immunity in a CD8^+^ T‐cell‐dependent manner, but it is worthwhile to note the elevated expression of immune checkpoints (especially PD‐1) (Figure 2J). We next investigated whether PGAM1 suppression could improve the antitumor efficacy of PD‐1 blockade. C57BL/6 mice bearing Hepa1‐6 subcutaneous tumors were treated with vehicle, anti‐PD‐1 mAb, KH3 or the combined treatment once every 3 days when the tumor size reached ≈ 100 mm^3^ (Figure S6E, Supporting Information). We observed that compared to the vehicle, anti‐PD‐1 and KH3 monotherapy inhibited tumor growth. Furthermore, the combination of KH3 and anti‐PD‐1 therapy further decreased tumor weight compared to any monotherapy alone (Figure 6D). Subsequently, we performed a parallel experiment with the same setup based on a genetic approach with shPgam1 (Figure S6F, Supporting Information), and the experiment showed consistent results. Hepa1‐6 subcutaneous tumor samples were harvested for further immunological validation. Immunofluorescence (IF) confirmed this promotion of CD8^+^ T‐cell infiltration (Figure S6G, Supporting Information). Flow cytometry was further performed to confirm comprehensive immune infiltration of different immune cell subpopulations, which indicated improved antitumor responsiveness in the combined treatment group. The KH3 monotherapy group was associated with increased infiltration of CD8^+^ T‐cells and a significant increase in the activated CD8^+^ T‐cell subset (IFN‐γ^+^ CD8^+^ T‐cells) compared to that in the vehicle group (Figure 6E,F; Figure S6H, Supporting Information). We also detected higher serum and tumor homogenate levels of HMGB1 in the KH3 monotherapy and combined groups (Figure S6I, Supporting Information). Next, we validated this combination therapeutic efficacy in Hepa16 orthotopic tumors (Figure S6J, Supporting Information). The combination of KH3 and anti‐PD‐1 mAb treatment also showed better antitumor efficacy (Figure 6G). Orthotopic tumor immune cell infiltration was also detected by flow cytometry and IF (Figure 6H,I; Figure S6K, Supporting Information), which revalidated the promoting tendency of CD8^+^ T‐cell infiltration. In addition, we also tested the tolerability and safety of KH3 or anti‐PD‐1 monotherapy and combined treatment in our HCC mouse model. The single or combined treatment was well tolerated, as we did not observe significant body weight loss (Figure S6L, Supporting Information) or obvious abnormalities of internal organs such as the spleen, kidney and heart (Figure S6M, Supporting Information) in subcutaneous and orthotopic models. With the above evidence, we demonstrate the efficacy and safety of KH3 and anti‐PD‐1 coblockade in several HCC preclinical models.

To further verify that this synergistic effect was dependent on CD8^+^ T‐cells mediated by PGAM1 inhibition, we depleted CD8^+^ T‐cells in a subcutaneous tumor model in C57BL/6 mice (Figure S6N, Supporting Information). The depletion of CD8^+^ T‐cells in tumors was confirmed by flow cytometry (Figure S6O, Supporting Information). We observed that anti‐CD8α mAb treatment significantly eliminated the difference in tumor burden between the shNC and shPgam1 groups and dampened anti‐PD‐1 therapeutic efficacy (Figure 6J). These results suggested that CD8^+^ T‐cells are major effector immune cells that dominate antitumor immunity induced by PGAM1 inhibition.

We next validated the correlation between PGAM1 expression and anti‐PD‐1 immunotherapy efficacy in HCC patients. We detected PGAM1, LCN2, CD8 and PD‐L1 expression by utilizing HCC samples from biopsies of 6 HCC patients before immunotherapy (Figure 6K). Compared with responders who achieved a partial response after the administration of anti‐PD‐1 immunotherapy, nonresponders who demonstrated an enlargement of tumor size had higher PGAM1 and LCN2 expression as well as lower CD8 expression (Figure 6L). These results suggested that HCC patients with low PGAM1 expression might benefit more from anti‐PD‐1 immunotherapy.

## 3 Discussion

Present studies on PGAM1 have mainly focused on the metabolic regulation of metabolism and molecular signaling pathways inside tumor cells. The immunomodulatory effect of PGAM1 has not yet been reported and remains to be elucidated. To confirm this, we applied high‐throughput mass cytometry to decipher the orthotopic immune landscape changes after PGAM1 inhibition. Targeting PGAM1 in HCC cells could reprogram “cold” tumors into “hot” tumors with an inflammatory TME that favors the infiltration of functional cytotoxic T‐lymphocytes (CTLs). Previous evidence has proven that aberrant tumor metabolism impairs the antitumor immune response. Glucose competition between tumor cells and CTLs and accumulation of toxic metabolites (e.g., lactate) can confine CTLs under nutrient‐deficient conditions and impair their viability as well as cytotoxic function, which favors immune escape.^[^ ^17^, ^21^ ^]^ In addition to this common principle, new evidence points to a critical role of ferroptosis in modifying the infiltration of immune cells and determining immunotherapeutic efficacy.^[^ ^22^ ^]^ Here, we screened PGAM1 as a key glucometabolic gene and an independent prognostic indicator for HCC patients. Our results confirmed that PGAM1 silencing inhibited the proliferation of HCC cells in vitro and tumor growth in vivo in a ferroptosis‐dependent manner and potentiated the infiltration of CD8^+^ T‐cells. We identified that targeting ferroptosis reversed the facilitation of CD8^+^ T‐cell infiltration rather than other T‐cell infiltration in PGAM1‐deficient HCC tumors in vivo. Several possible mechanisms were speculated in the present study, including the chemotaxis of DAMPs and downregulation of PD‐L1.

LCN2 was found to be the most significant DEG of the ferroptosis pathway after PGAM1 inhibition. Congruously, a recent study suggested that the leukemia inhibitory factor receptor (LIFR)‐NF‐κB‐LCN2 axis is involved in liver carcinogenesis and ferroptosis sensitivity.^[^ ^23^ ^]^ Another study showed that nuclear protein 1, transcriptional regulator (NUPR1)‐mediated LCN2 expression inhibits ferroptosis by diminishing iron accumulation and subsequent oxidative damage.^[^ ^24^ ^]^ Recently, LCN2 has emerged as a novel pleiotropic target responsible for various immune responses, inflammation, apoptosis, epithelial‐mesenchymal transition, tumor progression, invasion, metastasis, and energy metabolism.^[^ ^25^ ^]^ We then explored the mechanism by which PGAM1 inhibition decreases the expression of LCN2. KEGG analysis indicated that the PI3K‐AKT signaling pathway was the most significantly enriched (Figure 3B), which was validated by Western blotting, and several papers reported that LCN2 could be regulated by AKT.^[^ ^26^ ^]^

PGAM1 plays a critical role in cancer metabolism by critically catalyzing the conversion of 3‐PG to 2‐PG during aerobic glycolysis, which regulates intermediates used as precursors for anabolic biosynthesis. It is reasonable to hypothesize that PGAM1 inhibition led to energy stress, which was verified in our experiment (Figure 4K,L). Elevated levels of ROS could be caused by energy stress^[^ ^15^, ^27^ ^]^ and are a common phenotype of ferroptosis.^[^ ^20^ ^]^ In addition, previous research indicated that PGAM1 inhibitors induced ROS production, consequently abrogating p‐AKT to inhibit cell proliferation in NSCLC cells. Based on this evidence, we elucidated that targeting PGAM1 suppresses LCN2 expression via energy stress/ROS‐dependent inhibition of AKT.

PGAM1 interacts with ACTA2 mainly through residues 201–210 to facilitate cancer cell migration independent of its metabolic activity.^[^ ^7^ ^]^ Here, catalytically inactive H186R and Δ201–220 mutants lacking ACTA2 association were individually transfected into cells with stable knockdown of PGAM1 (Figure S7E, Supporting Information). We determined that decreased AKT expression and an optimized immune microenvironment by PGAM1 inhibition are mainly mediated through diminished enzymatic activity. Reportedly, tumors in which the PI3K/AKT pathway is abnormally activated are sensitive to immunotherapy.^[^ ^17^, ^18^, ^20^ ^]^ After the inhibition of AKT mediated by PGAM1 suppression, we confirmed that the expression of PD‐L1 in HCC was downregulated, which could promote the infiltration of CD8+ T‐cells since the inhibitory signals decreased. Although the present results indicate that the promotion of CD8+ T‐cell infiltration mediated by PGAM1 inhibition might be ferroptosis‐dependent, further work is warranted to experimentally explore the immunogenic mechanism of ferroptosis.

The most clinically significant finding of the present study is that it explored innovative strategies to overcome resistance to ICB, which is an urgent need. KH3 was established as an allosteric inhibitor of PGAM1 activity in a previous study by our collaborative team. Its therapeutic efficacy has already been proven for pancreatic cancer in several preclinical models.^[^ ^4c^ ^]^ Our results confirm that KH3 is an effective agent for anti‐HCC therapy in multiple preclinical models, including subcutaneous or orthotopic Hepa16 xenografts in immunodeficient or immunocompetent mice, as well as PDX models in NSG mice. We validated that KH3 could not only suppress HCC progression by inducing ferroptosis in a manner that is not inferior to sorafenib but also convert immune cold into inflamed tumors by potentiating robust T‐cell‐mediated antitumor immunity, which could sensitize HCC to anti‐PD‐1 blockade. Recently, several studies reported that ferroptosis is associated with HCC progression and antitumor immunity. Zhang et al. validated that mitochondrial translocator protein (TSPO) promotes HCC progression by inhibiting ferroptosis and promotes HCC immune escape by upregulating PD‐L1 expression through Nrf2‐mediated transcription.^[^ ^28^ ^]^ Another study reported that targeted xCT‐mediated ferroptosis and pro‐tumoral polarization of macrophages is effective against HCC and enhances the efficacy of the anti‐PD‐1/L1 Response.^[^ ^29^ ^]^ Moreover, we considered the potential toxicity or other side effects of KH3. Experiments such as weight monitoring, CBC and CMP analysis and HE is staining of vital organs were performed, which indicated the good safety of KH3. In addition, the clinical significance of PGAM1 expression was assessed in HCC patients receiving anti‐PD‐1 immunotherapy, and higher expression of PGAM1 was found in non‐responders. A diagnostic immunoassay based on circulating tumor‐associated autoantibodies, including the PGAM1 autoantibody, was recently established and proposed for advanced‐stage non‐small cell lung cancer (NSCLC) patients receiving PD‐1/L1 immunotherapy.^[^ ^30^ ^]^ Thus, further studies are required to validate the predictive value of circulating PGAM1 levels for immunotherapeutic efficacy in HCC patients.

In summary, we established the first proof of concept that targeting PGAM1 not only restrains HCC growth by promoting ferroptosis via energy stress/ROS‐dependent degradation of the AKT/LCN2 axis but also downregulates PD‐L1, thus potentiating robust CD8^+^ T‐cell‐mediated antitumor immunity and synergizing with anti‐PD‐1 immunotherapy (Figure 6M). These results suggest that PGAM1 is a potentially druggable target that “kills two birds with one stone”, considering both aberrant metabolism and the immune status of HCC; thus, this approach could address a major clinical conundrum in cancer immunotherapy.

## 4 Experimental Section

### In Vivo Experiments

All mice were housed in a specific pathogen‐free facility in the Laboratory Animal Center of Fudan University, with 5–6 mice per cage, according to the guidelines of the National Academy of Sciences and the National Institutes of Health. Permission of animal experiments was obtained from the Animal Care and Use Committee of Fudan University (DSF‐2020‐064). Mouse Hepa1‐6 HCC cells (4×10^6^ cells resuspended in 100 µL PBS) were subcutaneously injected to grow subcutaneous tumors in C57BL/6 mice (male, 6–8 weeks old, weighing ≈ 19–21 g). Subcutaneous tumor volumes were determined by measuring the length and width of the tumor with calipers and were calculated by (length × width^2^)/2. Animals were euthanized if they exhibited signs of distress or when maximum diameter of tumors reached > 1.5 cm. For the orthotopic tumor model, subcutaneous Hepa1‐6 tumors were cut into cubes (1 mm^3^) under aseptic conditions. Single cubes were then inoculated into the liver parenchyma of C57BL/6 mice anesthetized using 1% pentobarbital sodium. (For orthotopic injection of cells, 20ul Hepa1‐6 cells suspension mixed with Matrigel was prepared and inoculated into the liver via microinjector) Adequate analgesia was given during and after surgery.

### Clinical Tissue Samples

The HCC tissue samples of tissue microarray (TMA) were acquired at Zhongshan Hospital, Fudan University. All human samples were anonymously coded in accordance with local ethical guidelines (as stipulated by the Declaration of Helsinki). Written informed consent was obtained from each patient, and the study protocol was approved by the Review Board of Zhongshan Hospital, Fudan University (Y2022‐473).

### Statistical Analysis

To estimate the statistical significance of differences between the two groups, unpaired Student's *t* tests were used to calculate two‐tailed *p* values. Experimental results were shown as mean ± SD. A chi‐square test was performed to compare the categorical variables. Pearson's correlation analysis was performed to determine the correlation between two variables. Survival analysis was performed using KaplanMeier curves and evaluated with log‐rank tests. *p* values are labeled in figures. *p* values were denoted as follows: ^*^ *p* < 0.05, ^**^ *p* < 0.01, ^***^ *p* < 0.001, and ^****^ *p* < 0.0001. The mean fluorescence density was analyzed by ImageJ (NIH, 1.8.0). Statistical analyses were performed by using GraphPad Prism (version 8.0).

### HCC Dataset and Bioinformatic Analysis

The transcriptomic data of 159 HCC patients from Cell_ZS cohort were obtained in NODE (https://www.biosino.org/node) by pasting the accession (OEP000321) into the text search box or through the URL: https://www.biosino.org/node/project/detail/OEP000321. All the patients underwent primary curative resection from June 2010 to December 2014 at Zhongshan Hospital and received no prior anticancer treatments. The transcriptomic data of other HCC validation cohorts were obtained from GEO datasets (GSE14520 and GSE76427), ICGC datasets (LIRI‐JP, https://dcc.icgc.org/) and TCGA‐LIHC data (https://portal.gdc.cancer.gov/). Differentially expressed genes (DEGs) between C1 and C3 were analyzed by using “limma” package, with an adjusted *p* < 0.05 and |log2FC| ≥1. Single‐sample gene set enrichment analysis (ssGSEA) by utilizing “GSVA” package was separately conducted for each sample to generate infiltration scores for 24 immune cell clusters, calculating the proportion of TICs among different expression groups and assessing immune‐related biological functions. The immune gene sets were acquired from the MSigDB database (https://www.gsea‐msigdb.org/). Immune scores were generated by using xCell algorithm. The final ssGSEA score was defined as the larger value generated by one of two algorithms. The R packages used in the present study is listed in Table S4 (Supporting Information).

### Mass Cytometry and Data Analysis

Single‐cell suspension was prepared by the procedure mentioned above. For mass cytometry manipulation, purified antibodies in Table S5 (Supporting Information) were purchased from BioLegend, eBioscience, BioXcell, R&D systems, and BD Biosciences. Antibody labeling with the indicated metal tag was performed using the MaxPAR antibody Labeling kit (Fluidigm). Conjugated antibodies were titrated for optimal concentration before use. Cells were washed once with PBS and then stained with 100 µL of 250 nm cisplatin (Fluidigm) for 5 min to exclude dead cells, and then incubated in Fc receptor‐blocking solution before stained with surface antibodies cocktail for 30 min on ice. Cells were washed twice with FACS buffer (PBS+0.5%BSA) and fixed in 200 µL of intercalation solution (Maxpar Fix and Perm Buffer containing 250 nm 191/193Ir, Fluidigm) overnight. After fixation, cells were washed once with FACS buffer and then perm buffer (eBioscience), stained with intracellular antibodies cocktail for 30 min on ice. Cells were washed and resuspend with deionized water, adding into 20% EQ beads (Fluidigm), acquired on a mass cytometer (Helios, Fluidigm). CyTOF data analysis was performed as following: 1) Data from each sample were debarcoded from raw data using a doublet‐filtering scheme with unique mass‐tagged barcodes. 2) FCS file from each batch were normalized through bead normalization method. 3) Manually gate data using a FlowJo software to exclude debris, dead cells, and doublets, remain live, single immune cells. 4) Apply the X‐shift clustering algorithm to all cells to partition the cells into distinct phenotypes based on marker expression levels. 5) Annotate cell type of each cluster according to its marker expression pattern on a heatmap of cluster versus marker. 6) Use the dimensionality reduction algorithm t‐SNE to visualize the high‐dimensional data in two dimensions and show distribution of each cluster and marker expression and difference among different sample types. 7) Perform *t*‐test statistical analysis on the frequency of annotated cell population.

### Cell Culture and Transfection

Human HCC cell lines Hep3B, HCCLM3, MHCC97H, PLC/PRF/5, SNU398, SK‐Hep‐1, normal liver cell line L02, and mouse HCC cell line Hepa16 were obtained from Liver Cancer Institute, Fudan University (Shanghai, China). All cell lines have been authenticated by short tandem repeat (STR) profiling and validated to be mycoplasma‐negative. All cells were cultured in Dulbecco's modified Eagle's medium (Gibco) supplemented with 10% heat‐inactivated fetal bovine serum (Gibco), 1% penicillin, and streptomycin (Gibco) at 37°C in a humidified incubator containing 5% CO2. Hepa16, PLC/PRF/5, and SNU398 cells with stable PGAM1 knockdown, and Hep3B cells with stable PGAM1 overexpression were achieved by lentiviral infection. All cell lines underwent selection in the presence of 5 µg ml^−1^ puromycin. PGAM1 plasmid was purchased from Shanghai Genechem Co., Ltd. LCN2 and Lcn2 plasmid was purchased from Guangzhou RiboBio Co., Ltd. Wild‐type PGAM1 plasmid, Δ201220 (201220 aa deleted) and H186R PGAM1 mutants were kindly gifted from Prof. M Huang (State Key Laboratory of Drug Research, Chinese Academy of Sciences, Shanghai, China). For siRNA transfection, cells were plated at 30%−60% confluence in OPTI‐MEM serum‐free medium and transfected with a specific siRNA duplex using Lipofectamine RNAiMAX Reagent Agent (Life Technologies) according to the manufacturer's instructions. siRNAs against ACTA2 were siACTA2 pool purchased from Santa Cruz. For plasmid transfections, cells were grown to 60% confluence in 6‐cm dishes and transfected with 4 µg of plasmids using 4 µL of Lipofectamine 3000 (Invitrogen) according to the manufacturer's instructions. For experiments using PGAM1 truncations (Δ201220), MG‐132 at 10 µm was added at 6 h prior to the harvest of the cells. The oligo sequences of shRNA were listed in Table S6 (Supporting Information).

### RNA Extraction and Real‐Time Quantitative PCR (qPCR)

Total RNAs were extracted using TRIZOL reagent (Invitrogen). Real‐time qPCR was performed using the StepOnePlus Real‐time PCR system (Applied Biosystems) with SYBR Green PCR Master Mix (Takara, China) according to the manufacturer's protocol. The sequences of primers for PCR were listed in Table S7 (Supporting Information).

### Antibody and Reagents

The antibodies used in Western blotting, immunohistochemistry, immunofluorescence, and in vivo experiments are listed in Table S8 (Supporting Information). The ELISA kits and other reagents were also listed.

### Flow Cytometry Analysis

Fresh mouse tumors were digested with the Mouse Tumor Dissociation Kit (Miltenyi). Dissociated tumor samples were filtered through the 70 µm strainer and then disposed by 36% percoll reagent to remove cell debris. ACK Lysis Buffer was used to lyse the erythrocytes. Then cells were blocked with Fc block (anti‐mouse CD16/32, BioLegend) on ice for 30 min. The samples were stained for lymphoid and myeloid immune populations with the following antibodies listed in Table S9 (Supporting Information). For the assessment of intracellular markers, cells were incubated with a Cell Stimulation Cocktail (BioLegend, 423 303) and stained with anti‐mouse IFN‐γ after fixation/permeabilization.

Other experimental information is presented in Supporting Information.

## Conflict of Interest

The authors declare no conflict of interest.

## Author Contributions

Y.Z., Y.W., Z.L., and J.W. contributed equally to this work. Y.Z., Y.W., Z.L., and J.W. performed the experiments. L.J. synthesized and identified KH3. D.S., C.W., and C.G. analyzed the data and drafted figures. G.S., J.Z., and J.F. provided the human samples. Y.W. wrote the manuscript. L.Z., A.K., and J.C. conceived the project and revised the manuscript.

## Supporting information

Supporting Information

## Data Availability

The data that support the findings of this study are available from the corresponding author upon reasonable request.
