# Supplementary material for: PGAM1 Inhibition Promotes HCC Ferroptosis and Synergizes with Anti‐PD‐1 Immunotherapy
Source: Adv Sci (Weinh). 2023 Sep 14;10(29):2301928. doi: 10.1002/advs.202301928 (PMC10582428; doi:10.1002/advs.202301928)
Supplement: Supplementary file 1 — Supporting Information [file ADVS-10-2301928-s001.pdf]

ADVANCED SCIENCE Open Access

Supporting Information

for *Adv. Sci.*, DOI 10.1002/advs.202301928

PGAM1 Inhibition Promotes HCC Ferroptosis and Synergizes with Anti-PD-1 Immunotherapy

*Yimin Zheng, Yining Wang, Zhou Lu, Jinkai Wan, Lulu Jiang, Danjun Song, Chuanyuan Wei, Chao Gao, Guoming Shi, Jian Zhou, Jia Fan, Aiwu Ke**, *Lu Zhou** *and Jiabin Cai**

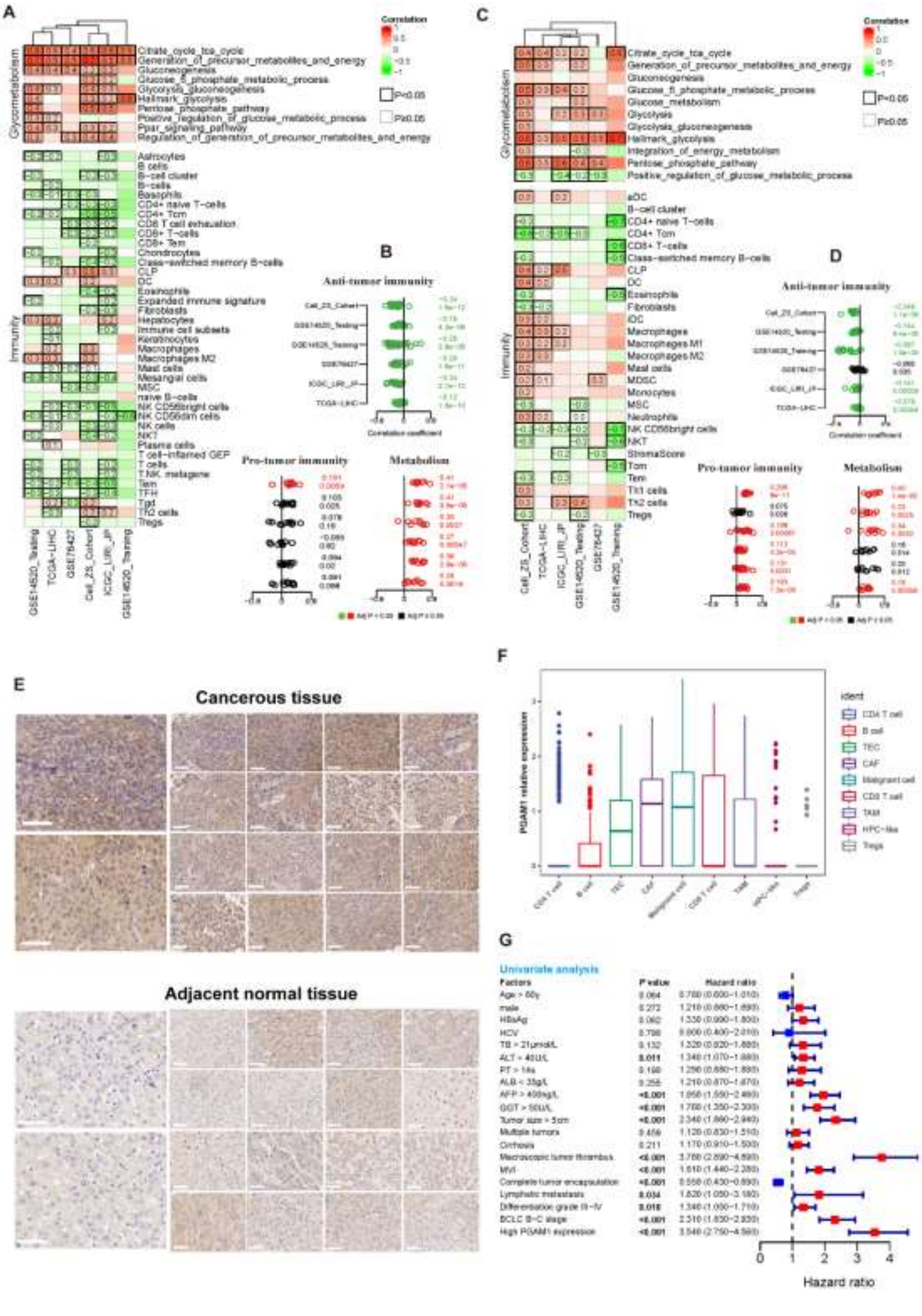

A
B
C
D
E
F
G
Anti-tumor immunity
Pro-tumor immunity
Metabolism
Cancerous tissue
Adjacent normal tissue
Glycometabolism
Immunity
Correlation
P<0.05
P≥0.05
PGAM1 relative expression
Univariate analysis
Factors
P value
Hazard ratio
Age > 60y
male
HBsAg
HCV
Cirrhosis
Microvascular tumor thrombus
MVI
Complete tumor encapsulation
Lymphatic metastasis
Differentiation grade III~IV
BCLC B~C stage
High PGAM1 expression
Hazard ratio

Figure S1. PGAM1 is a novel immunometabolic target correlated with poor prognosis in HCC.

(A-B) Correlation heatmap **(A)** and forest plot **(B)** for MDH1 expression, anti-tumor immunity, pro-tumor immunity and metabolism based on five HCC datasets.

(C-D) Correlation heatmap **(C)** and forest plot **(D)** for PGK1 expression, anti-tumor immunity, pro-tumor immunity and metabolism based on five HCC datasets.

(E) Representative IHC pictures of PGAM1 expression levels in corresponding tissue from patients with HCC shown in Fig. 1F. (Scale bars=100μm)

(F) Relative expression of PGAM1 on different cell subsets of 9 HCC samples based on single-cell-seq dataset from GSE125449.

(G) Univariate Cox regression analysis for clinopathological characteristics correlated to overall survival of HCC patients in the Zhongshan TMA cohort.

Abbreviation: MDH1, malate dehydrogenase 1; PGK1, phosphoglycerate kinase 1; HBsAg, hepatitis B surface antigen; HCV, hepatitis C virus; TB, total bilirubin; ALB, albumin; ALT, alanine aminotransferase; GGT, gamma-glutamyltransferase; PT, prothrombin time; AFP, alpha-fetoprotein; MVI, microvascular invasion; BCLC, Barcelona Clinic Liver Cancer.

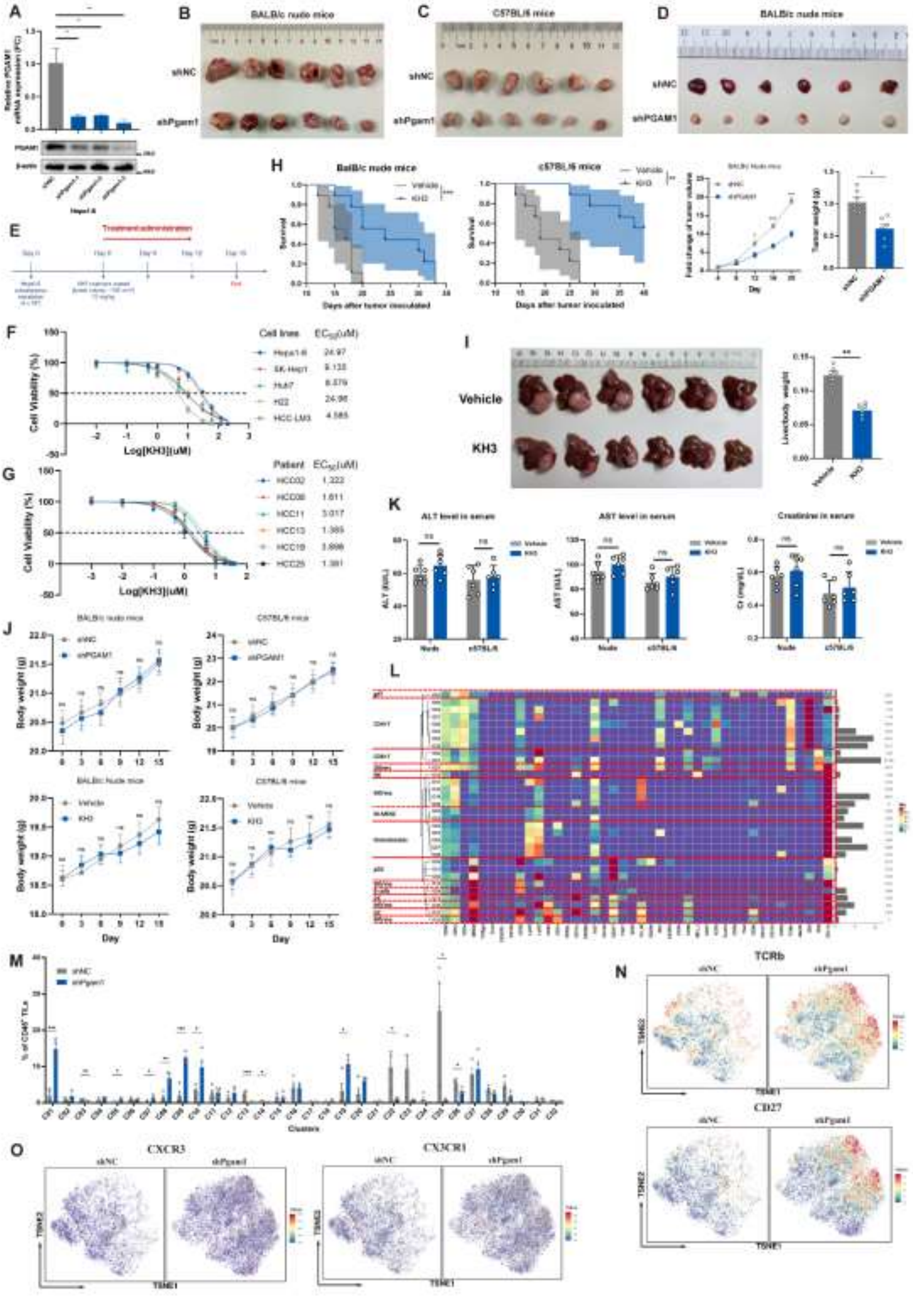
A
B
BALB/c nude mice
shNC
shPgam1
C
C57BL/6 mice
D
BALB/c nude mice
shNC
shPGAM1
E
Treatment administration
F
Cell lines
Hepa1-6
SK-Hep1
HuH7
H22
HCC-LM3
24.97
9.135
8.579
24.96
4.585
Cell Viability (%)
Log[KH3](uM)
G
Patient
HCC02
HCC08
HCC11
HCC13
HCC19
HCC25
1.322
1.611
3.017
1.389
3.898
1.381
Cell Viability (%)
Log[KH3](uM)
H
BalB/c nude mice
C57BL/6 mice
Vehicle
KH3
Survival
Days after tumor inoculated
I
Vehicle
KH3
J
BALB/c nude mice
C57BL/6 mice
shNC
shPGAM1
Vehicle
KH3
Body weight (g)
Day
K
ALT level in serum
AST level in serum
Creatinine in serum
Vehicle
KH3
Nude
c57BL/6
L
M
shNC
shPgam1
Clusters
N
TCRb
shNC
shPgam1
TSNE1
TSNE2
CD27
O
CXCR3
CX3CR1
shNC
shPgam1
TSNE1
TSNE2

Figure S2. Targeting PGAM1 inhibits HCC progression and favors the antitumor immune response by reshaping the immune microenvironment in HCC.

(A) The relative mRNA expression and protein expression of Hepa1-6 cells transfected with negative control (shNC) or shRNA targeting Pgam1 (shPgam1-1/2/3). The Hepa1-6 cell line treated with shPgam1-3 was chosen as shPgam1 Hepa1-6 cells in the subsequent experiments (The mRNA expression of the other 3 group were normalized according to the result of shNC).

(B-C) Tumor image in immunodeficient nude mice **(B)** or immunocompetent C57BL/6 mice **(C)** with subcutaneous inoculation of shNC or shPgam1 Hepa1-6 cells (n=6 per group).

(D) Tumor growth curve, tumor weight and tumor image in immunodeficient nude mice with subcutaneous inoculation of shNC or shPGAM1 PLC/PRF/5 cells (n=6 per group).

(E) KH3 treatment strategy for HCC growth inhibition in subcutaneous Hepa1-6 xenografts (n=6 per group). Six days after Hepa1-6 cell inoculation, KH3 was intraperitoneally injected (75 mg/kg) once every 3 days for 3 times. Mice were sacrificed at day 15.

(F) EC_{50} curve of KH3 suppressing the proliferation of 3 human HCC cell lines and 2 murine HCC cell lines.

(G) EC_{50} curve of KH3 suppressing the proliferation of HCC primary cells isolated from 6 patients.

(H) Kaplan−Meier curves of overall survival of nude mice and C57BL/6 mice with subcutaneous inoculation of Hepa1-6 cells treated vehicle (PLGA) or KH3 (n=9 per group, endpoint: diameter of the tumor beyond 18mm or cachexia).

(I) Final images and liver/body weight (%) of C57BL/6 mice with orthotopic injection of Hepa1-6 cells treated with vehicle (PLGA) or KH3 (n=6 per group).

(J) Body weight in nude mice and C57BL/6 mice with subcutaneous inoculation of shNC and shPgam1 Hepa1-6 cells (upper panel) or inoculation of Hepa1-6 cells treated vehicle (PLGA) or KH3(lower panel) (n=6 per group).

(K) Serum level of ALT, AST and creatinine in nude mice and C57BL/6 mice with subcutaneous inoculation of Hepa1-6 cells treated vehicle (PLGA) or KH3 (n=6 per group).

(L) The heatmap demonstrating the normalized expression levels of 42 markers in 32 immune subsets within the total $CD45^+$ tumor-infiltrating leukocyte population.

(M) Histogram for the frequencies of 32 subdivided immune cell subsets within the $CD45^+$ population.

(N) Density *t*-SNE plot for the TCRb and CD27 expression of infiltrated $CD8^+$ T cell within the $CD45^+$ population of shNC and shPgam1 Hepa1-6 orthotopic tumors by CyTOF analysis.

(O) Density *t*-SNE plot for the expression of chemokine receptor (CXCR3 and CX3CR1) in infiltrated $CD8^+$ T cell within the $CD45^+$ population of shNC and shPgam1 Hepa1-6 orthotopic tumors by CyTOF analysis.

The data were presented as the means ± SD of three independent experiments or triplicates. *P* values were determined by a two-tailed unpaired *t* test. *$P < 0.05$; **$P < 0.01$; ***$P < 0.001$; n.s., not significant, $P > 0.05$. **Abbreviation:** *t*-SNE, *t*-distributed stochastic neighbor embedding; TCRb, T cell receptor β; CXCR3, CXC motif chemokine receptor 3; CX3CR1, C-X3-C motif chemokine receptor 1.

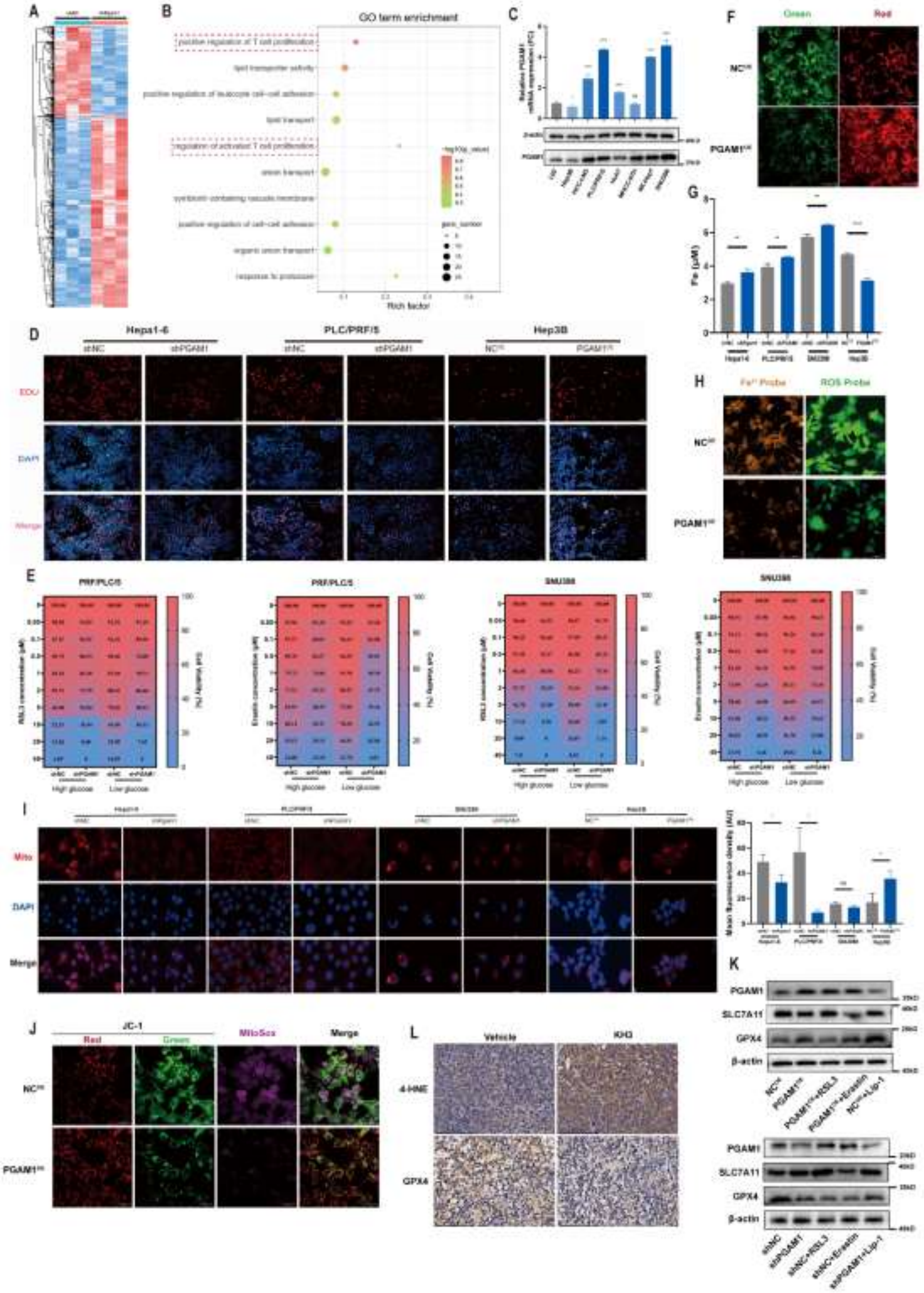

A
B
GO term enrichment
Rich factor
C
D
Hepa1-6
PLC/PRF/5
Hep3B
shNC
shPGAM1
EDU
DAPI
Merge
E
PRF/PLC/5
SNU398
High glucose
Low glucose
F
Green
Red
G
Fe (μM)
H
Fe2+ Probe
ROS Probe
I
Mito
J
JC-1
MitoSox
K
PGAM1
SLC7A11
GPX4
β-actin
L
Vehicle
KH3
4-HNE

Figure S3. PGAM1 inhibition enhances ferroptosis in HCC cells.

(A) Heatmap for DEGs of shNC and shPgam1 Hepa1-6 subcutaneous tumors in RNA-seq. “DESeq2” package，difference threshold: $|\log2FC| \geq 1$ & adj. $P < 0.05$.

(B) GO enrichment analysis for DEGs in RNA-seq.

(C) Relative mRNA expression and protein level of PGAM1 in 7 human HCC cell lines and the L-02 normal liver cell line (The mRNA expression of the other 7 HCC cell lines were normalized according to the result of L-02).

(D) The proliferation capability and statistics of three HCC cell lines was detected by EdU assay (Scale bars=100μm).

(E) Heatmap demonstrated targeting PGAM1 enhanced the sensitivity to two ferroptosis inducers (RSL3, Erastin) in PLC/PRF/5 and SNU398 cell lines under high glucose and low glucose media.

(F) BODIPY 581/591 C11 fluorescent probe showing decreased lipid peroxidation accumulation in Hep3B cells with PGAM1 overexpressed (scale bars=50μm).

(G) total iron was detected in PGAM1-silencing Hepa1-6, PLC/PRF/5, SNU398 cell lines and PGAM1 overexpressed Hep3B cell line. The protein concentration of each cellular lysis was titrated to 500ug/ul.

(H) FerroOrange and ROS probe exhibiting decreased intracellular Fe^{2+} levels and ROS levels in Hep3B cells with PGAM1 overexpressed (Scale bars=50μm).

(I) Mitochondrial membrane potential detection and statistics in PGAM1-silencing Hepa1-6, PLC/PRF/5, SNU398 cell lines and PGAM1 overexpressed Hep3B cell lines (scale bars=50μm).

(J) JC-1 detections with mitoSOX staining comprehensively showing ROS decrease and enhanced membrane potential in mitochondria in Hep3B cells with PGAM1 overexpressed (scale

bars=50μm).

(K) Western blot showing the expression of SLC7A11 and GPX4 in PGAM1-silencing PLC/PRF/5 cell line and PGAM1 overexpressed Hep3B cell line treated with RSL3, Erastin or liproxstatin-1.

(L) IHC examining the level of 4-HNE and the expression of GPX4 in mouse subcutaneous tumor tissues of Hepa1-6 cells treated with vehicle (PLGA) or KH3 (scale bars=100μm).

The data were presented as the means ± SD of three independent experiments or triplicates. *P* values were determined by a two-tailed unpaired *t* test. *$P < 0.05$; **$P < 0.01$; ***$P < 0.001$; ****$P < 0.0001$; n.s., not significant, $P > 0.05$. **Abbreviation:** FC, fold change; GO, gene ontology. 4-HNE, 4-hydroxynonenal; SLC7A11, cystine/glutamate antiporter xCT; GPX4, glutathione peroxidase 4.

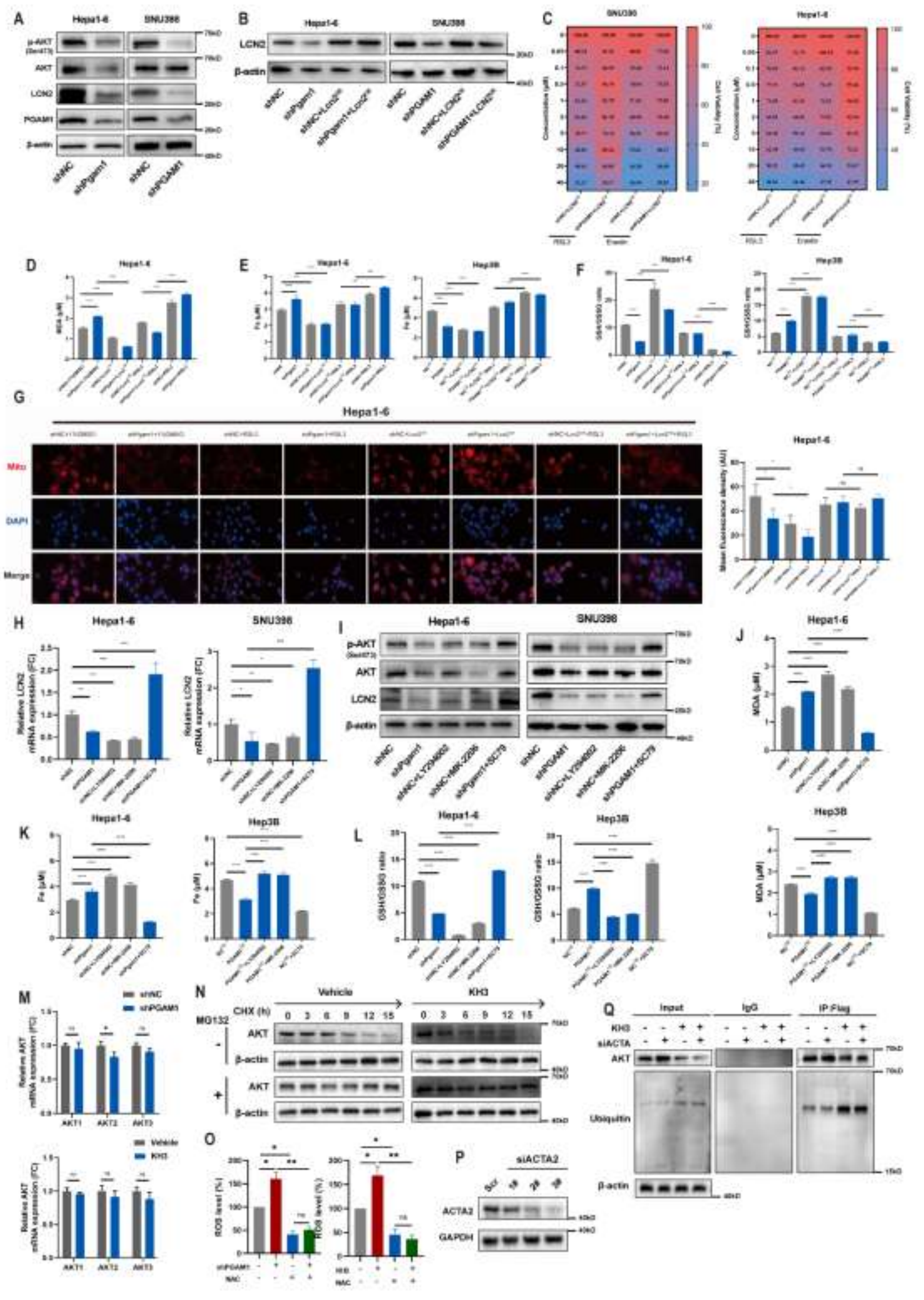
A
Hepa1-6
SNU398
p-AKT
AKT
LCN2
PGAM1
β-actin
shNC
shPgam1
shPGAM1
B
Hepa1-6
SNU398
LCN2
β-actin
C
SNU398
Hepa1-6
D
Hepa1-6
E
Hepa1-6
Hep3B
F
Hepa1-6
Hep3B
G
Hepa1-6
Mito
DAPI
Merge
H
Hepa1-6
SNU398
I
Hepa1-6
SNU398
p-AKT
(Ser473)
AKT
LCN2
β-actin
shNC
shPgam1
shNC+LY294002
shNC+MK-2206
shPgam1+SC79
shPGAM1
shPGAM1+SC79
J
Hepa1-6
K
Hepa1-6
Hep3B
L
Hepa1-6
Hep3B
Hep3B
M
shNC
shPGAM1
AKT1
AKT2
AKT3
Vehicle
KH3
N
Vehicle
KH3
CHX (h)
0
3
6
9
12
15
MG132
AKT
β-actin
O
P
siACTA2
ACTA2
GAPDH
Q
Input
IgG
IP:Flag
KH3
siACTA
AKT
Ubiquitin
β-actin

Figure S4. Targeting PGAM1 suppresses LCN2 expression via energy stress/ROS-dependent inhibition of AKT.

(A) Western blot detected the protein expression of PGAM1, LCN2, AKT, p-AKT in indicated Hepa1-6 and SNU398 cell lines.

(B) Western blot validated the establishment of the above 2 HCC cell lines with recombinant LCN2 expression.

(C) Heatmap demonstrated recombinant LCN2 expression reversed the enhancement of ferroptosis sensitivity induced by PGAM1 inhibition in the above 2 HCC cell lines under high glucose condition.

(D-F) The intracellular concentration of MDA **(D)**, total iron **(E)** and GSH/GSSG ratio **(F)** were detected in indicated Hepa1-6 and Hep3B cell lines treated with RSL3 (2 μM) in the presence or absence of recombinant LCN2 expression. The protein concentration of each cellular lysis was titrated to 500ug/ul.

(G) Mitochondrial membrane potential detection and statistics in indicated Hepa1-6 cells treated with RSL3 (2 μM) in the presence or absence of recombinant Lcn2 expression (scale bars=50μm).

(H) Relative mRNA expression of LCN2 in the above 2 HCC cell lines treated with LY294002 (15 μM), MK-2206 (15 μM) or SC79 (15 μM).

(I) Western blot detected the protein expression of LCN2, AKT, p-AKT in indicated Hepa1-6 and SNU398 cell lines treated with LY294002 (15 μM), MK-2206 (15 μM) or SC79 (15 μM).

(J-L) The intracellular concentration of MDA, total iron and GSH/GSSG in indicated Hepa1-6 and Hep3B cell lines treated with RSL3 (2 μM) in the presence or absence of recombinant LCN2 expression. The protein concentration of each cellular lysis was titrated to 500ug/ul (n=3).

(M) Relative mRNA expression of three AKT isoforms in PLC/PRF/5 cells and Hepa1-6 cells with the indicated modified PGAM1 or KH3 treatments.

(N) Western blotting showing the effect of KH3 on AKT stability in Hepa1-6 cells incubated with CHX or MG132 at the indicated time points.

(O) PLC/PRF/5 cells and Hepa1-6 cells were treated with genetic and pharmacological inhibition (10uM KH3) of PGAM1 respectively, 10 mM NAC, or a combination of PGAM1 inhibition and NAC for 6 h. The levels of ROS were examined by ROS Assay Kit.

(P) Protein expression of PLC/PRF/5 cells transfected with scramble (Scr) or small interfering RNA targeting ACTA2 (siACTA2-1/2/3). siACTA2-3 was chosen in the subsequent experiments.

(Q) Co-IP analyses were conducted to identify the function of KH3 (pharmacological PGAM1 inhibition) and ACTA2 on the AKT ubiquitination level in Hepa1-6 cells incubated with MG132.

The data were presented as the means ± SD of three independent experiments or triplicates. *P* values were determined by a two-tailed unpaired *t* test. *$P < 0.05$; **$P < 0.01$; ***$P < 0.001$; ****$P < 0.0001$; n.s., not significant, $P > 0.05$. **Abbreviation:** MDA, malondialdehyde; GSH, glutathione; GSSG, oxidized glutathione; NAC, N-acetyl-l-cysteine.

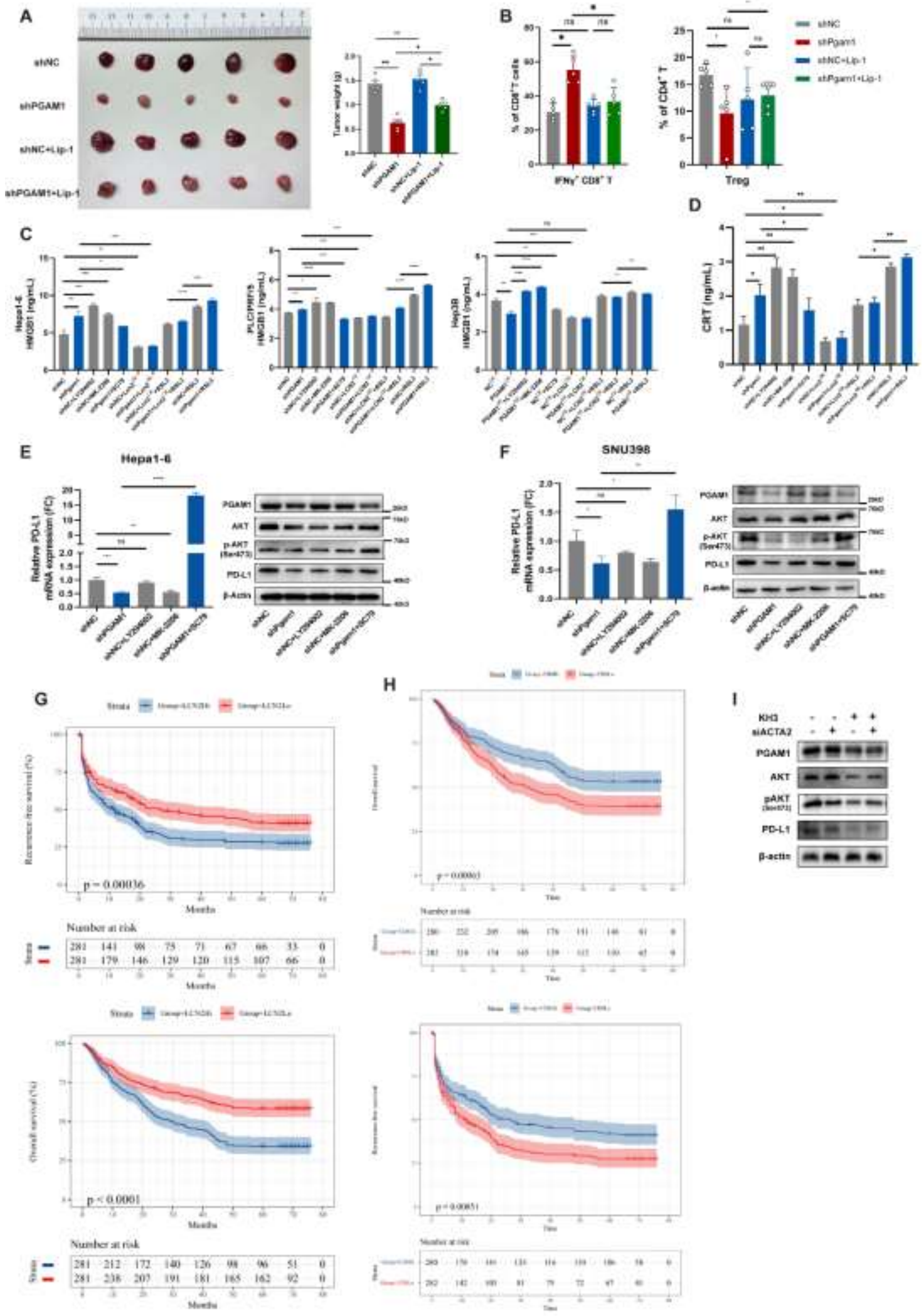
A
shNC
shPGAM1
shNC+Lip-1
shPGAM1+Lip-1
Tumor weight (g)
B
% of CD8+T cells
IFNγ+ CD8+ T
% of CD4+ T
Treg
shNC
shPgam1
shNC+Lip-1
shPgam1+Lip-1
C
Hepa1-6 HMGB1 (ng/mL)
PLC/PRF/5 HMGB1 (ng/mL)
Hep3B HMGB1 (ng/mL)
D
CRT (ng/mL)
E
Hepa1-6
Relative PD-L1 mRNA expression (FC)
PGAM1
AKT
p-AKT (Ser473)
PD-L1
β-Actin
F
SNU398
Relative PD-L1 mRNA expression (FC)
PGAM1
AKT
p-AKT (Ser473)
PD-L1
β-actin
G
Strata
Number at risk
Months
p = 0.00036
p < 0.0001
H
Number at risk
Time
I
KH3
siACTA2
PGAM1
AKT
pAKT (Ser473)
PD-L1
β-actin

Figure S5. PGAM1 inhibition could promote CD8$^+$ T cell infiltration and down regulate PD-L1 in HCC.

(A) Tumor weight and tumor images of PLC/PRF/5 subcutaneous xenografts of ferroptosis inhibition experiment (n=5 per group).

(B) The percentage of IFNγ$^+$ CD8$^+$ T cells and Treg cells in ferroptosis inhibition experiment.

(C). The expression level of cellular supernatant HMGB1 in indicated PLC/PRF/5, Hep3B and Hepa1-6 cell lines treated with LY294002 (15 μM), MK-2206 (15 μM), SC79 (15 μM) or RSL3 (2 μM) in the presence or absence of recombinant LCN2 expression.

(D) The expression level of cellular supernatant CRT in Hepa1-6 cell line treated with LY294002 (15 μM), MK-2206 (15 μM), SC79 (15 μM) or RSL3 (2 μM) in the presence or absence of recombinant LCN2 expression.

(E-F) Relative mRNA of PD-L1 as well as protein expression of PGAM1, AKT, p-AKT and PD-L1 in indicated SNU398 and Hepa1-6 cells treated with LY294002 (15 μM), MK-2206 (15 μM) or SC79 (15 μM); The mRNA expression of the other 4 group were normalized according to the result of shNC.

(G) Kaplan-Meier overall survival and recurrence-free survival for HCC tissues with LCN2^{Hi} and LCN2^{Lo} in the Zhongshan TMA cohort.

(H) Kaplan-Meier overall survival and recurrence-free survival for HCC tissues with CD8^{Hi} and CD8^{Lo} in the Zhongshan TMA cohort.

(I) Western blotting showing the effect of PGAM1 inhibition and/or ACTA2 depletion on the expression of AKT, p-AKT and PD-L1 in Hepa1-6 cells.

The data were presented as the means ± SD of three independent experiments or triplicates. *P*

values were determined by a two-tailed unpaired t test. *$P < 0.05$; **$P < 0.01$; ***$P < 0.001$; ****$P < 0.0001$; n.s., not significant, $P > 0.05$. **Abbreviation:** Lip-1, liproxstatin-1; Treg, regulatory T cells.

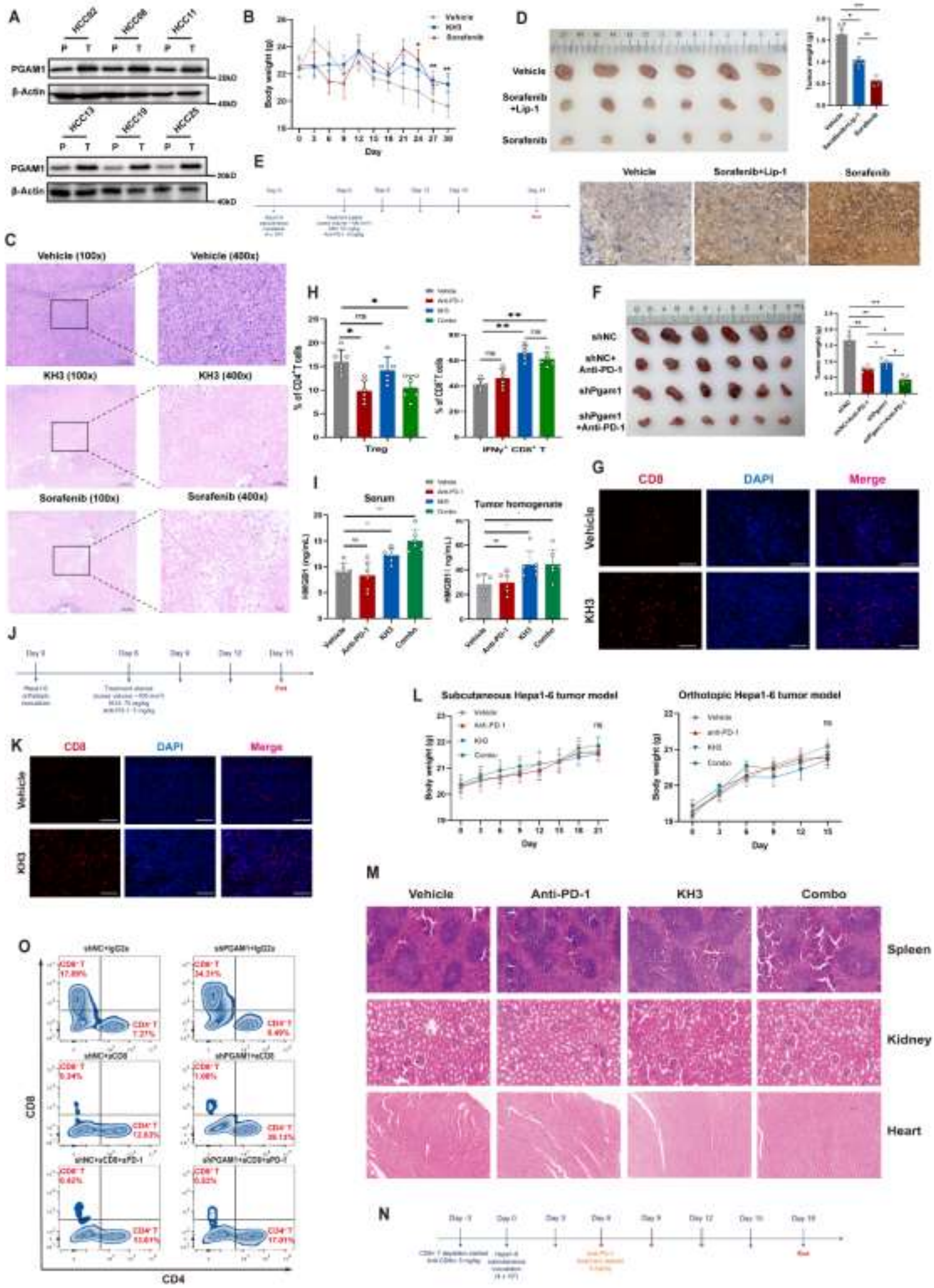
A
HCC02
HCC08
HCC11
P T P T P T
PGAM1
β-Actin
HCC13
HCC19
HCC25
B
Vehicle
KH3
Sorafenib
Body weight (g)
Day
D
Vehicle
Sorafenib
+Lip-1
Sorafenib
Tumor weight (g)
E
Vehicle
Sorafenib+Lip-1
Sorafenib
C
Vehicle (100x)
Vehicle (400x)
KH3 (100x)
KH3 (400x)
Sorafenib (100x)
Sorafenib (400x)
H
Treg
IFNγ+ CD8+ T
F
shNC
shNC+
Anti-PD-1
shPgam1
shPgam1
+Anti-PD-1
I
Serum
Tumor homogenate
HMGB1 (ng/mL)
Vehicle
Anti-PD-1
KH3
Combo
G
CD8
DAPI
Merge
Vehicle
KH3
J
K
CD8
DAPI
Merge
Vehicle
KH3
L
Subcutaneous Hepa1-6 tumor model
Orthotopic Hepa1-6 tumor model
Body weight (g)
Day
M
Vehicle
Anti-PD-1
KH3
Combo
Spleen
Kidney
Heart
O
CD8
CD4
N

Figure S6. KH3 exhibits significant antitumor effects in PDX models and synergizes with PD-1 blockade immunotherapy.

(A) Immunoblotting of PGAM1 and β-Actin in HCC (T) and para-tumor normal tissues (P) from 6 patients.

(B) Body weight of the PDX models treated with vehicle (PLGA), KH3 or Sorafenib (n=6 per group).

(C) Representative H&E staining images of indicated treatment groups mentioned above.

(D) Endpoint tumor images, tumor weight and IHC results of 4-HNE of the PDX models treated with vehicle (PLGA), sorafenib, or a combination of sorafenib and liproxstatin-1 (n=6 per group).

(E) Combination treatment strategy for HCC growth inhibition in subcutaneous Hepa1-6 xenografts (n=6 per group). anti-mouse PD-1 antibodies and/or KH3 were intraperitoneally injected (7.5 mg/kg and 75 mg/kg, respectively) once every 3 days for 4 times.

(F) Tumor weight and tumor images of subcutaneous Hepa1-6 tumors treated with genetic inhibition of Pgam1, anti-PD-1 mAb, vehicle or shPgam1 combined with anti-PD-1 mAb (n=6 per group).

(G) Representative IF images of $CD8^+$ T cells in subcutaneous Hepa1-6 tumors treated with vehicle (PLGA) or KH3 (scale bars=200μm).

(H) Quantification of $IFN\gamma^+$ $CD8^+$ T cells and Treg cells of subcutaneous Hepa1-6 tumors treated with vehicle (PLGA) or KH3 combined with IgG2a or anti-PD-1 mAb.

(I) The HMGB1 level of serum and tumor homogenate of subcutaneous Hepa1-6 tumors treated with vehicle (PLGA) or KH3 combined with IgG2a or anti-PD-1 mAb.

(J) Combination treatment strategy for orthotopic Hepa1-6 growth inhibition.

(K) Representative IF images of $CD8^+$ T cells in orthotopic Hepa1-6 tumors treated with vehicle (PLGA) or KH3 (scale bars=200μm).

(L) Body weight of C57BL/6 mice with subcutaneous and orthotopic tumors in the combination therapy experiment.

(M) Representative H&E staining images of the spleen, kidney and heart of indicated treatment groups are demonstrated.

(N) Schematic strategy of $CD8^+$ T cell depletion of shNC and shPgam1 subcutaneous Hepa1-6 xenografts. Mice were intraperitoneally injected with either IgG2a or αCD8 mAb (5mg/kg) on the indicated days (blue arrows). Six days after tumor cell inoculation, tumor bearing mice were treated with either vehicle, αPD-1 mAb (5mg/kg) on the indicated days (orange arrows).

(O) Representative images of $CD8^+$ tumor-infiltrating leukocytes analyzed by flow cytometry for subcutaneous Hepa1-6 tumors treated with anti-CD8 mAb combined with IgG2a or anti-PD-1 mAb.

Data in Figure B, F, H, I were presented as the mean ± SD; P values were determined by one-way ANOVA in Fig. B; P values were determined by two-way ANOVA in Figure F, H, I; $^*P < 0.05$; $^{**}P < 0.01$; $^{***}P < 0.001$; n.s., not significant, $P > 0.05$. **Abbreviation:** IFN-γ, interferon gamma; Treg, regulatory T cells; H&E, hematoxylin-eosin.

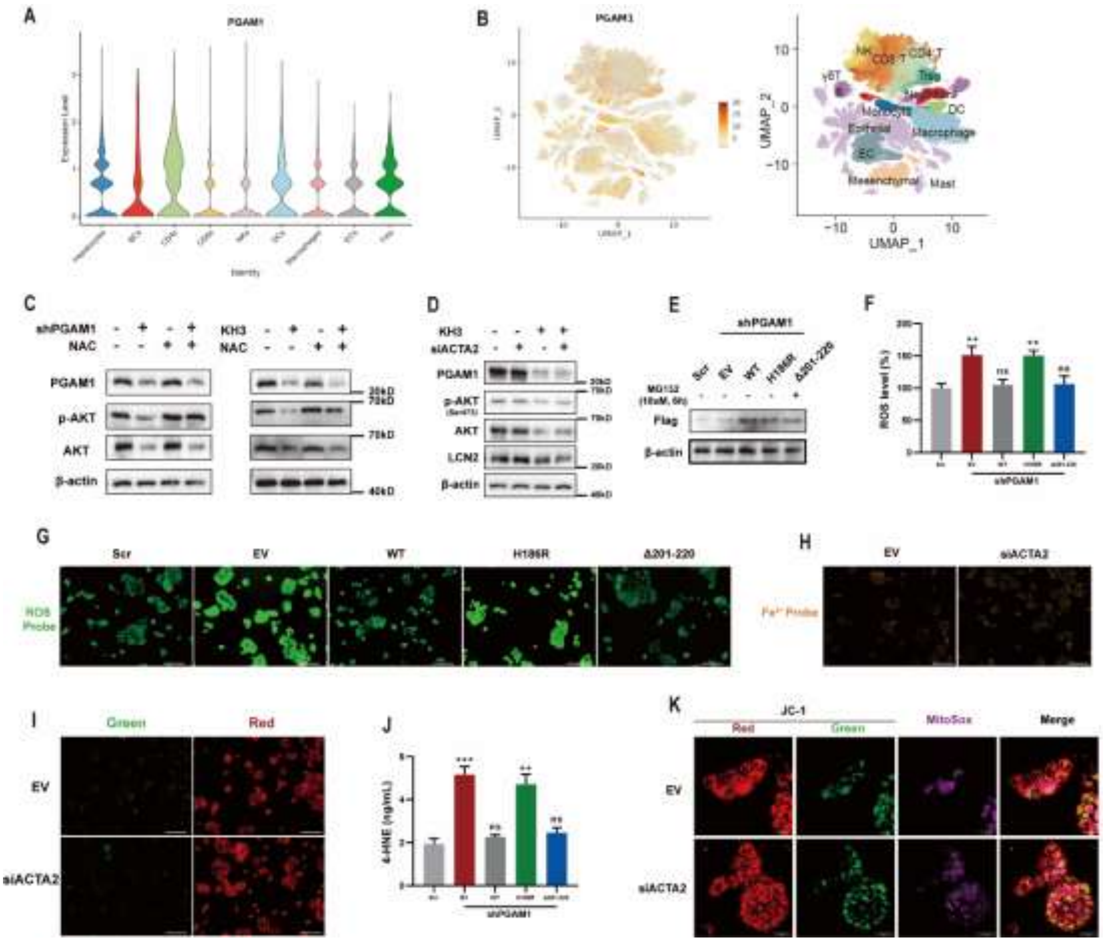
A
PGAM1
Expression Level
Identity
B
PGAM1
UMAP_1
UMAP_2
NK
CD8 T
CD4 T
Treg
γδT
Monocyte
DC
Epithelial
Macrophage
EC
Mesenchymal
Mast
C
shPGAM1
NAC
KH3
PGAM1
p-AKT
AKT
β-actin
20kD
70kD
40kD
D
KH3
siACTA2
PGAM1
p-AKT
AKT
LCN2
β-actin
E
shPGAM1
Scr
EV
WT
H186R
Δ201-220
MG132 (10uM, 6h)
Flag
β-actin
F
ROS level (%)
shPGAM1
G
Scr
EV
WT
H186R
Δ201-220
ROS Probe
H
EV
siACTA2
I
Green
Red
EV
siACTA2
J
4-HNE (ng/mL)
shPGAM1
K
JC-1
Red
Green
MitoSox
Merge
EV
siACTA2

Figure S7. Experiments related to Figure 1 and Figure 4.

(A) Relative expression of PGAM1 on different cell subsets of 10 HCC samples based on single-cell-seq dataset from GSE149614.

(B) Expression of PGAM1 on different cell types as shown by UMAP plot of 79 HCC samples based on single-cell-seq dataset from web (http://meta-cancer.cn:3838/scPLC).

(C) Western blotting showing the effect of genetic or pharmacological PGAM1 inhibition (10uM KH3 treatment for 48h) and/or ROS elimination (NAC treatment for 48 h) on the expression of p-AKT, AKT in PLC/PRF/5 and Hepa1-6 cells respectively.

(D) Western blotting showing the effect of PGAM1 inhibition (10uM KH3 treatment for 48h) and/or ACTA2 depletion on the expression of p-AKT, AKT and LCN2 in Hepa1-6 cells.

(E) Immunoblotting showed the expression level of reconstituted PGAM1 wildtype or mutants in PLC/PRF/5 cells.

(F-G) Quantitation of ROS level and ROS detection in PGAM1 stably depleted PLC/PRF/5 cells reconstituted with PGAM1 wildtype or indicated mutants.

(H) FerroOrange probe showed ACTA2 depletion exerted no difference of intracellular Fe^{2+} levels in PLC/PRF/5 cells.

(I) BODIPY 581/591 C11 (a marker of lipid peroxidation) fluorescent probe showing no difference of lipid peroxidation between EV and siACTA2 group in PLC/PRF/5 cells.

(J) Quantitation of 4-HNE level in PGAM1 stably depleted PLC/PRF/5 cells reconstituted with PGAM1 wildtype or indicated mutants.

(K) JC-1 detections with mitoSOX staining comprehensively showed ACTA2 depletion exerted no difference of ROS level or membrane potential in mitochondria in PLC/PRF/5 cells.

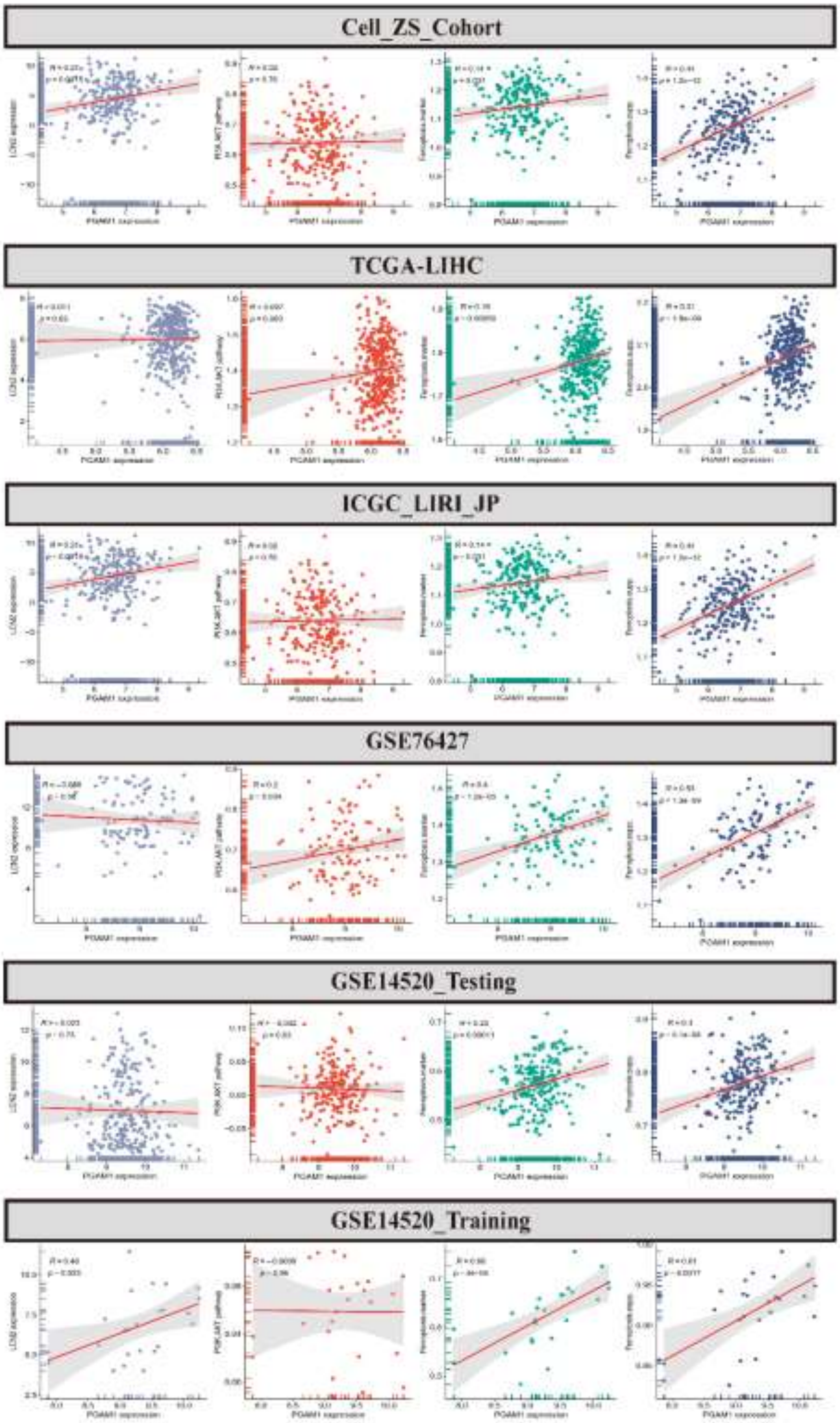

Figure S8. Correlation analysis of PGAM1 with LCN2, PI3K-AKT signaling pathway, ferroptosis marker gene and ferroptosis suppressor gene in five HCC datasets. Coefficient (r) was determined by Pearson's correlation.

Table S1. Univariate and multivariate Cox regression analyses for 5-year OS.

Factors	Univariate analysis (95%CI)	P value	Multivariate analysis (95%CI)	P value
Age >60 y	0.78 (0.60-1.01)	0.064		
Male	1.21 (0.86-1.69)	0.272		
HBsAg	1.33 3(0.99-1.80)	0.062		
HCV	0.90 (0.40-2.01)	0.790		
TB > 21μmol/L	1.32 (0.92-1.88)	0.132		
ALB < 35g/L	1.21 (0.87-1.67)	0.255		
ALT > 40U/L	1.34 (1.07-1.68)	**0.011**	1.03 (0.81-1.32)	0.785
GGT > 50U/L	1.76 (1.35-2.30)	**<0.001**	1.16 (0.86-1.57)	0.322
AFP > 400ng/ml	1.95 (1.55-2.46)	**<0.001**	1.54 (1.20-1.99)	**<0.001**
Cirrhosis	1.17 (0.91-1.50)	0.211		
Tumor size > 5cm	2.34 (1.86-2.94)	**<0.001**	1.54 (1.2-1.98)	**<0.001**
Multiple tumors	1.12 (0.83-1.51)	0.459		
Complete tumor encapsulation	0.55 (0.43-0.69)	**<0.001**	0.81 (0.63-1.04)	0.100
Macroscopic tumor thrombus	3.76 (2.89-4.89)	**<0.001**	1.69 (1.10-2.61)	**0.018**
Microvascular invasion	1.81 (1.44-2.28)	**<0.001**	1.17 (0.91-1.49)	0.213
Lymphatic metastasis	1.82 (1.05-3.18)	**0.034**	1.57 (0.89-2.76)	0.120
Differentiation grade III-IV	1.34 (1.05-1.71)	**0.018**	1.10 (0.85-1.40)	0.473
BCLC stage B+C	2.31 (1.83-2.93)	**<0.001**	1.28 (0.88-1.87)	0.201
High PGAM1 expression	3.54 (2.75-4.56)	**<0.001**	3.11 (2.40-4.04)	**<0.001**

Table S2. Detailed markers for 32 immune subsets of the CyTOF analysis.

Cell type	Cluster	Markers
$CD8^+$ T	C01	$CD38^{lo}$ $Ly6C^+$ $Ki67^+$ $PD1^+$ $CD8^+$ $CD3e^+$
$CD8^+$ T	C02	$Ly6C^+$ $CD8^+$ $CD3e^+$
gdT	C03	$CD44^+$ $CD3e^+$ $CD69^{lo}$ $TCRgd^{lo}$
$CD4^+$ T	C04	$CD44^+$ $CD3e^+$ $MHCII^{lo}$ $Ly6C^{lo}$ $Ki67^+$ $TCRb^+$ $CD4^+$ $CD11b^+$
$CD4^+$ T	C05	$MHCII^{lo}$ $Ki67^{lo}$ $CCR2^+$ $TCRb^+$ $CD4^+$

CD4$^+$ T (Treg)	C06	CD3e$^+$ Ki67$^+$ CD25$^+$ ICOS$^+$ TCRb$^+$ CD4$^+$
CD4$^+$ T	C07	CD3e$^+$ Ly6C$^+$ TCRb$^+$ CD4$^+$
CD4$^+$ T	C08	CD44^{lo} CD3e$^+$ CD38$^+$ PD1$^+$ CD86$^+$ TCRb$^+$ CD4$^+$
CD4$^+$ T	C09	CD44$^+$ CD3e$^+$ Ki67$^+$ PD1$^+$ TCRb$^+$ CD4$^+$
CD4$^+$ T	C10	CD44^{lo} CD3e$^+$ TCRb$^+$ CD4$^+$
DC	C11	MHCII$^+$ CD11c$^+$ CD11b$^+$ CD11c^{lo}
DC	C12	CD44$^+$ MHCII$^+$ CD38$^+$ CD64$^+$ CD11c$^+$ CD317$^+$ F4_80$^+$ CD80^{lo} CD11b$^+$
Macro/Mono	C13	CD44^{lo} MHCII$^+$ CD38$^+$ CD64$^+$ CD14$^+$ F4_80$^+$ CD11b$^+$
Macro/Mono	C14	MHCII$^+$ CD38$^+$ CD11b$^+$
NK	C15	CD11c$^+$ NK1_1$^+$ CD11b$^+$
Macro/Mono	C16	MHCII$^+$ Ly6C$^+$ CD64$^+$ Ki67$^+$ CD86^{lo} CD11b$^+$
Macro/Mono	C17	CD44$^+$ Ly6G$^+$ Ly6C$^+$ Ki67$^+$ F4_80$^+$ CD11b$^+$
Macro/Mono	C18	CD44$^+$ Ki67^{lo} F4_80$^+$ CCR2$^+$ CD11b$^+$
Macro/Mono	C19	CD44$^+$ Ki67^{lo} F4_80$^+$ CD11b$^+$
Macro/Mono	C20	CD44$^+$ CD11c^{lo} F4_80$^+$ CD11b$^+$
Others	C21	CD44$^+$ CD38^{lo} Ly6C$^+$ Ki67$^+$ PD1^{lo} CD8$^+$ CD11b$^+$
M-MDSC	C22	CD11b$^+$ Ly6C$^+$
M-MDSC	C23	Ki67$^+$ CD11b$^+$ Ly6C$^+$
Granulocytes	C24	Ly6G$^+$ Ly6C$^+$ PD1$^+$ CD11b$^+$
Granulocytes	C25	Ly6G$^+$ Ly6C$^+$ CD14$^+$ CD11b$^+$
Granulocytes	C26	Ly6G$^+$ Ly6C$^+$ CD11b$^+$
Granulocytes	C27	Ly6G$^+$ Ly6C$^+$ Ki67$^+$ CD11b$^+$
Granulocytes	C28	Ly6G$^+$ Ly6C$^+$ Ki67^{lo} CCR2^{lo} CD11b$^+$
B cells	C29	MHCII$^+$ CD38$^+$ B220^{lo}
pDC	C30	MHCII^{lo} CD38$^+$ CD317$^+$ CD206$^+$ CD11b$^+$
pDC	C31	MHCII$^+$ Ki67^{lo} CD317$^+$ CD11b^{lo}
pDC	C32	MHCII$^+$ Ly6C$^+$ CD11c$^+$ CD317$^+$

Table S3. NSG Mouse Hematology and Biochemistry.

NSG mice (n=6) were treated with either vehicle control or Sorafenib or KH3 for 30 days. CBC and CMP (Comprehensive metabolic panel) analysis shows no significant

difference in the hematopoietic and biochemical properties among the three groups of mice.

Test Name (units)	Reference range (low-high)	Vehicle day30 (mean)	30mg/kg Sorafenib day30 (mean)	75mg/kg KH3 day30 (mean)
**Hematology**				
WBC (x $10^3/\mu L$)	2.6-10.1	7.45 ± 5.23	6.83 ± 4.27	7.34 ± 3.98
LYM (x $10^3/\mu L$)	0.3-1.8	0.92 ± 0.68	0.88 ± 0.34	0.76 ± 0.23
MONO (x $10^3/\mu L$)	0-0.3	0.21 ± 0.18	0.16 ± 0.09	0.17 ± 0.12
GRAN (x $10^3/\mu L$)	0.4-2.0	1.35 ± 1.05	1.14 ± 0.83	1.34 ± 0.53
LYM (%)	0-99.9	12.3 ± 3.05	9.32 ± 2.15	10.56 ± 3.67
MONO (%)	0-99.9	5.08 ± 1.35	3.28 ± 1.14	4.89 ± 1.37
GRAN (%)	0-99.9	21.42 ± 7.05	17.53 ± 6.87	19.37 ± 8.45
RBC (x $10^6/\mu L$)	6.5-10.1	5.36 ± 2.41	6.87 ± 4.67	8.47 ± 3.98
MCV (fl)	42.3-55.9	53.10 ± 7.25	48.65 ± 9.35	50.25 ± 6.78
HCT (%)	32.8-48	27.45 ±14.04	33.18 ±16.53	29.47 ±13.27
MCH (pg)	13.7-18.1	18.15 ± 2.64	15.18 ± 3.76	17.67 ± 2.79
MCHC (g/dL)	29.5-35.1	32.85 ± 3.65	34.76 ± 5.87	34.88 ± 6.19
RDWR (%)	0-99.9	15.32 ± 3.32	17.37 ± 4.76	17.66 ± 3.79
RDWA (%)	0-99.9	34.31 ± 3.92	40.37 ± 5.90	38.94 ± 6.18
PLT (x $10^3/\mu L$)	250-1540	321.00 ± 78.69	467.34 ± 98.36	387.87± 74.98
MPV (fl)	0-99.9	6.72 ± 1.37	9.46 ± 2.65	8.67 ± 3.44
HGB (g/dL)	10-16.1	8.11 ± 3.39	12.76 ± 4.78	11.77 ± 3.56
**Biochemistry**				
Na (mmol/L)	115-191	155 ± 28	149 ± 25	160 ± 22
K (mmol/L)	5.2-9.7	6.3 ± 1.9	5.8 ± 1.7	6.3 ± 2.1
Cl (mmol/L)	8.5-114	65 ± 24	72 ± 19	87 ± 32
Ca (mg/dL)	9.2-10.4	9.81 ± 0.45	9.47 ± 0.41	9.77 ± 0.36
P (mg/dL)	4.8-11.1	7.92 ± 2.45	6.72 ± 1.33	8.14 ± 2.24
Fe (mg/dL)	200-282	241 ± 25	263 ± 27	258 ± 31
Cr (mg/dL)	0.23-0.70	0.56 ± 0.14	0.59 ± 0.16	0.52 ± 0.19
BUN (mg/dL)	10.3-29.1	24.2 ± 5.34	26.5 ± 6.25	25.2 ± 5.76
GLU (mg/dL)	62-132	97 ± 19	104 ± 21	112 ± 25
TB (mg/dL)	0.35-0.62	0.48 ± 0.09	0.54 ± 0.11	0.51 ± 0.13
DB (mg/dL)	0.05-0.24	0.15 ± 0.04	0.16 ± 0.03	0.18 ± 0.08
ALT (IU/L)	0-135.8	58.8 ± 20.4	64.3 ± 25.8	64.9 ± 22.4
AST (IU/L)	35.7-135.7	85.7 ± 16.3	87.4 ± 19.2	90.7 ± 19.4
ALP (IU/L)	71.2-85	78.1 ± 4.5	80.1 ± 5.3	79.4 ± 6.3
TP (g/dL)	5.5-6.3	5.8 ± 0.22	5.6 ± 0.27	5.8 ± 0.26
ALB (g/dL)	2.5-2.9	2.7 ± 0.12	2.7 ± 0.18	2.8 ± 0.13
TC (mg/dL)	63.5-92.5	81.3 ± 8.3	86.4 ± 10.2	79.1 ± 9.4

Table S4. R packages used in the bioinformatic exploration for PGAM1.

R packages	Version	Source
circlize	0.4.13	CRAN
clusterProfiler	4.2.2	Bioconductor
ComplexHeatmap	2.11.0	Bioconductor
enrichplot	1.14.1	Bioconductor
GGally	2.1.2	CRAN
ggplot2	3.3.5	CRAN
ggpubr	0.4.0	CRAN
ggrepel	0.9.1	CRAN
GSVA	1.42.0	Bioconductor
igraph	1.2.11	CRAN
limma	3.50.0	Bioconductor
msigdbr	7.4.1	CRAN
psych	2.1.9	CRAN
survival	3.2-13	CRAN
survminer	0.4.9	CRAN
survMisc	0.5.5	CRAN
xCell	-	https://github.com/dviraran/xCell

Table S5. The detailed information of purified monoclonal antibody used in the CyTOF analysis.

No.	Label	Antibody	Clone	Vender	Location
1	89Y	CD45	30-F11	BioLegend	surface
2	113In	CD44	IM7	BioLegend	surface
3	115In	CD3e	145-2C11	BioLegend	surface
4	141Pr	MHC II	M5/114.15.2	BioLegend	surface
5	142Nd	TCRgd	GL3	BioXcell	surface
6	143Nd	Tim-3	RMT3-23	BioLegend	surface
7	144Nd	CX3CR1	SA011F11	BioLegend	surface
8	145Nd	CD163	S15049I	BioLegend	surface
9	146Nd	CD38	90	BioLegend	surface
10	147Sm	Ly6G	IA8	BioLegend	surface
11	148Nd	Ly6C	HK1.4	BioLegend	surface
12	149Sm	CD64/FcγRI	X54-5/7.1	BioLegend	surface
13	150Nd	CD14	Sa14-2	BioLegend	surface

14	151Eu	CD62L	MEL-14	BioLegend	surface
15	152Sm	CD11c	N418	BioLegend	surface
16	153Eu	CXCR5	L138D7	BioLegend	surface
17	154Sm	Ki-67	SolA15	BioLegend	intra
18	155Gd	CD103	2E7	BioLegend	surface
19	156Gd	CD317/BST2	44E9R	BioLegend	surface
20	157Gd	TIGIT	2190A	BioLegend	surface
21	158Gd	CD45R/B220	RA3-6B2	BioLegend	surface
22	159Tb	F4/80	C1:A3-1	Bio-Rad	surface
23	160Gd	CD206	C068C2	BioLegend	intra
24	161Dy	CD279/PD1	29F.1A12	BioLegend	surface
25	162Dy	CXCR3	CXCR3-173	BioLegend	surface
26	163Dy	CD25/IL-2R	3C7	BioLegend	surface
27	164Dy	CD86	GL-1	R&D systems	surface
28	165Ho	CD161c/NK1.1	PK136	BioLegend	surface
29	166Er	CD27	LG.3A10	BioLegend	surface
30	167Er	ICOS	C398.4A	BioLegend	surface
31	168Er	FoxP3	FJK-16s	eBioscience	intra
32	169Tm	CD69	H1.2F3	BioLegend	surface
33	170Er	Tbet	4B10	BioLegend	intra
34	171Yb	CD80	16-10A1	eBioscience	surface
35	172Yb	CD127/IL-7Ra	A7R34	BioLegend	surface
36	173Yb	CD115	AFS98	BioLegend	surface
37	174Yb	CD192/CCR2	475301	BioXcell	surface
38	175Lu	TCRb	H57-597	BD Biosciences	surface
39	176Yb	MertK	ZB10C42	BioLegend	surface
40	197Au	CD4	RM4-5	BioLegend	surface
41	198Pt	CD8	53-6.7	BioLegend	surface
42	209Bi	CD11b	M1/70	BioLegend	surface

Table S6. Oligo sequences for short hairpin RNA

Name	Species	Sequences (5’-3’)
**shRNA**		
shNC	Human	CCGGTTCTCCGAACGTGTCACGTCTCGAGACGTGA CACGTTCGGAGAATTTTTG
shPGAM1	Human	CCGGTTGCGAGTGCTTTGTTTACTACTCGAGTAGT AAACAAAGCACTCGCAATTTTTG
shNC	Mouse	CCGGTTCTCCGAACGTGTCACGTCTCGAGACGTGA CACGTTCGGAGAATTTTTG
shPgam1-1	Mouse	CCGGCGTCTATGAACTGGACAAGAACTCGAGTTCT

		TGTCCAGTTCATAGACGTTTTTG
shPgam1-2	Mouse	CCGGCCCTTCTGGAATGAAGAAATTCTCGAGAATT TCTTCATTCCAGAAGGGTTTTTG
shPgam1-3	Mouse	CCGGCCCTAGAAGGTTGGGATCAATCTCGAGATTG ATCCCAACCTTCTAGGGTTTTTG

Table S7. Specific primers for quantitative real-time PCR

Name		Sequences (5'-3')
PGAM1	F	GTGCAGAAGAGAGCGATCCG
	R	CGGTTAGACCCCCATAGTGC
Pgam1	F	AGCGACACTATGGCGGTCT
	R	TGGGACATCATAAGATCGTCTCC
ACTB	F	CCTTCCTGGGCATGGAGTC
	R	TGATCTTCATTGTGCTGGGTG
Actb	F	TGCTGTCCCTGTATGCCTCTG
	R	TGATGTCACGCACGATTTCC
LCN2	F	ACCAAGGAGCTGACTTCGGA
	R	TCAGCCGTCGATACACTGGT
Lcn2	F	GACTCAACTCAGAACTTGATCCCT
	R	AGCTCTGTATCTGAGGGTAGCTGT
AKT1	F	ATGAGCGACGTGGCTATTGT
	R	TGAAGGTGCCATCATTCTTG
AKT2	F	AACACAAGGAAAGGGAACCA
	R	AGGAGGCACCGTGGACA
AKT3	F	GCTCAGAGGGGAGTCATCAT
	R	GGAAGTATCTTGGCCTCCAG
PD-L1	F	TGTACCGCTGCATGATCAG
	R	AGTTCATGTTCAGAGGTGACTG
Pd-l1	F	TGCTGTCACTTGCTACGG
	R	ATCTTCCTTTTCCCAGTACACC

Table S8. Antibodies, ELISA kit and reagents

Name	Source	Identifier
**Antibodies**		
Anti-PGAM1	Cell Signaling Technology	12098S
Anti-PGAM1	Proteintech	16126-1-AP

Anti-AKT	Cell Signaling Technology	4691S
Anti-p-AKT	Cell Signaling Technology	4060S
Anti-PD-L1	Proteintech	66248-1-Ig
Anti-GPX4	Abcam	ab125066
Anti-SLC7A11	OriGene	TA301518
Anti-4-HNE	R&D Systems	MAB3249
Anti-AMPK	Cell Signaling Technology	2532S
Anti-p-AMPK	Cell Signaling Technology	2535S
Anti-LCN2	Abcam	ab125075
Anti-LCN2	Abcam	ab216462
Anti-LCN2	Proteintech	26991-1-AP
Anti-ACTA2	Abcam	ab124964
Anti-ubiquitin	Abcam	ab140601
Anti-CD8	Cell Signaling Technology	98941S
Anti-Beta Actin	Proteintech	66009-1-Ig
Goat anti-mouse IgG (H+L), HRP conjugate	Proteintech	SA00001-1
Goat anti-rabbit IgG (H+L), HRP conjugate	Proteintech	SA00001-2
Donkey anti-Rabbit IgG (H+L), Alexa Fluor 488	Invitrogen	A-21206
InVivoMAb IgG2a isotype	BioXcell	BE0089
InVivoMAb anti-mouse PD-1	BioXcell	BE0146
InVivoMAb anti-mouse CD8α	BioXcell	BE0004–1
**ELISA kit**		
Human CRT	Abcam	ab283995
Mouse CRT	Abcam	ab284624
4-HNE	Abcam	ab238538
Mouse CXCL10	Wellbio	EM3203S
Human HMGB1	JiangLai	JL13693
Mouse HMGB1	JiangLai	JL13702
Lipid peroxidation MDA	Beyotime	S0131S
GSH/GSSG	Beyotime	S0053
Total iron	Elabscience	E-BC-K139-M
**Reagents**		
CCK8	Yeasen	40203ES60
EdU	Beyotime	C0078S

Mito-Tracker Red CMXRos	Beyotime	C1035
RSL3	Selleck	S8155
Erastin	Selleck	S7242
Liproxstatin-1	Selleck	S7699
LY294004	Selleck	S1105
MK-2206	Selleck	S1078
SC79	Selleck	S7863
**Ferroptosis assays**		
FerroOrange	Dojindo	F374
BDP 581/591 C11	Dojindo	L267
ROS Assay Kit	Dojindo	R253
JC-1 MitoMP Detection Kit	Dojindo	MT09
mtSOX Deep Red	Dojindo	MT14

Table S9. Antibodies used for flow cytometry analysis

Name	Source	Identifier
Anti-CD45	BioLegend	103138
Anti-CD3	BioLegend	100220
Anti-CD4	BioLegend	100563
Anti-CD8	BioLegend	100705
Anti-CD45R/B220	BioLegend	103235
Anti-NK1.1	BioLegend	108705
Anti-CD25	BioLegend	101909
Anti-Foxp3	Invitrogen	126403
Anti-IFN-γ	BioLegend	505809
Anti-CD11b	BioLegend	101205
Anti-Ly6G	BioLegend	127617
Anti-Ly6C	BioLegend	128011
Anti-F4/80	BioLegend	123115
Zombie NIR™ Fixable Viability Kit	BioLegend	423105

Supplementary materials and Methods

Metabolomic analysis: The levels of metabolites in HCC cells were measured by liquid chromatography-mass spectrometry (LC–MS) analysis. Tumor cells (5×106/sample) were treated with extraction liquid (V methanol: V water=4:1) to extract metabolites. Samples were centrifuged at 15000 rpm for 15 min at 4 °C, and supernatants were collected and subjected to vacuum freeze-drying. All LC–MS analyses were performed with high-performance liquid chromatography (HPLC) equipped with a LC-20AB pump (Shimadzu, Japan). The 4000 QTRAP system was coupled to a mass spectrometer (AB Sciex, Framingham, MA) operated in multiple reaction monitoring (MRM) mode. Extracts were redissolved in 500 μL of extraction liquid (V methanol: V water=1:9) and separated by a Luna NH2 column (50 × 2.0 mm, 5 μm, Phenomenex). The binary solvent system consisted of mobile phase A (300 μL of acetic acid [HAc], 1.25 mL of NH4OH, 0.77 g of NH4OAc, 25 mL of ACN and 500 mL of water) and mobile phase B (ACN). The analysis program was as follows: 0.1 min, 85% B; 3 min, 30% B; 12 min, 2% B; 15 min, 2% B; and 16–28 min, 85% B. The following parameter settings were implemented: electrospray voltage, 5 kV; gas 1, 30; gas 2, 30; curtain gas, x`25; and temperature, 500. ProteoWizard was applied for peak integration, and the in-house MS2 database (BiotreeDB) was used for metabolite annotation.

Western blotting: Cells or tissues were lysed in RIPA lysis buffer containing protease inhibitors and phosphatase inhibitors (Biosharp) by vortexing vigorously for 2-3 sec followed by boiling for 15 min. Protein concentrations were determined using

a BCA assay (Thermo Scientific). Proteins were subjected to SDS-PAGE separation and transferred to PVDF membranes (Immobilon-P, Millipore). Membranes were blocked protein free rapid blocking buffer (Epizyme, PS108P) for 20 min at room temperature and blotted with primary antibodies at 4°C overnight. After washing with TBST three times for 30 min, membranes were incubated with horseradish peroxidase-conjugated anti-rabbit IgG (dilution, 1:5000) or anti-mouse IgG (dilution, 1:5000) antibodies at room temperature for 1 h. The membranes were washed with TBST for three times before visualized using Western Blotting Luminal Reagent (Beyotime) and subsequent exposure to Tanon Film (Tanon, Shanghai). All experiments were performed at least three independent times.

RNA extraction and real-time quantitative PCR (qPCR) : Total RNAs were extracted using TRIZOL reagent (Invitrogen). Real-time qPCR was performed using the StepOnePlus Real-time PCR system (Applied Biosystems) with SYBR Green PCR Master Mix (Takara, China) as previously described. The sequences of primers for PCR were listed in **Supplementary Table 6**.

Co-immunoprecipitation : Total cell lysates were prepared by incubating cells with NP-40 lysis buffer (20 mM Tris-HCl pH 8.0, 150 mM NaCl, 1% Nonidet P40, and 2 mmol L-1 EDTA) supplemented with a protease inhibitor cocktail (Roche) on ice for 30 min. Lysates were centrifuged at 14,000 g for 15 min. Protein concentration of the supernatants was measured using BCA protein assay kit (Beyotime). Anti-flag M2 beads (Sigma; A2220) were incubated with the supernatants for 2 hr at 4°C. To test the interaction between AKT and ubiquitin, anti-AKT antibody (CST; 4691S) was

incubated with the supernatants for 4 hr at 4°C, and then added the Protein A/G PLUS-Agarose (Santa Cruz; sc-2003) to incubate 2 more hr at 4°C. The beads were collected and washed six times with cold lysis buffer NP40. The bound proteins were eluted by boiling with 1x loading buffer containing 0.2% sodium dodecyl sulfate and 100 mM DTT. The supernatant was collected and separated using SDS-PAGE.

Cell viability, EdU assay: Cell viability was assayed by using a CCK8 kit (Yeasen Biotechnology, Shanghai, Co., Ltd.). In brief, cells were seeded into 96-well plates ($2*10^3$ cells/100 μl per well) and incubated with the indicated treatments. Subsequently, 90 uL fresh medium (without fetal bovine serum) containing 10 uL CCK-8 solutions was added to each well and incubated for 2 h (37°C, 5% CO_2). Absorbance at 450 nm was measured using a microplate reader (Cytation 5 Cell Imaging Multi-Mode Reader). The DNA synthesized rate was detected with EdU assay kit (Beyotime, C0078S) according to the manufacturer’s instructions.

Transmission electron microscopy (TEM): Hepa1-6 cells were plated in 10cm cell dish and then pretreated with RSL3 for 48 h. Next, cells were collected and fixed with TEM fixer (Servicebio, G1102). TEM imaging was conducted by Servicebio, Wuhan, China.

Transcriptomic sequencing (RNA-seq): Total RNA was extracted from Hepa1-6 cells (shNC and shPgam1) using TRIzol reagent (Invitrogen) according to the manufacturer’s instructions. RNA integrity was further validated using an Agilent Bioanalyzer 2100 (Agilent Technologies). Stranded RNA sequencing libraries were prepared by using the KAPA Stranded RNA-Seq Library Preparation Kit (Illumina)

following the manufacturer's instructions. RNA sequencing was performed on a HiSeq 4000 (Illumina). DEG analysis was achieved by using "DESeq2" package. Kyoto Encyclopedia of Genes and Genomes (KEGG) enrichment analysis and Gene Ontology (GO) analysis were performed to evaluate the gene sets enriched in RNA-seq data.

Immunohistochemistry (IHC), immunofluorescence staining (IF) and multiplex immunofluorescence assay (mIF): Formalin-fixed, paraffin-embedded tumor sections (5 μm thick) from different tumors were stained using anti-PGAM1, anti-LCN2 and anti-CD8 antibodies. The detailed IHC procedure has been previously described [46]. Tumor sections and staining were performed by Outdo Biotech. (Shanghai, China). For PGAM1 and LCN2 staining, samples with no staining in any tumor cells were defined as score 0, weakly stained samples as scored 1, moderately stained samples as scored 2, and strongly stained samples as scored 3. We also calculated the number of positive cells from five high magnification fields chosen at random as well as their mean intensities. For CD8 staining, we estimated the counts of cells with a strong intensity (brown staining) of membrane staining at 200× magnification. At least four fields were reviewed for each slide by two independent investigators in a randomized, double-blind manner. IF and mIF were utilized to investigate the expression of PGAM1, LCN2, CD8 and PD-L1 in samples, as described previously.

PGAM1 enzyme assay: PGAM1 activity was measured by multiple enzymes coupled assay. PGAM1 enzyme mix containing 100 mM Tris-HCl, 100 mM KCl, 5 mM

MgCl2, 1 mM ADP, 0.2 mM NADH, 5 mg/ml recombinant PGAM1, 0.5 units/ml enolase, 0.5 units/ml recombinant pyruvate kinase M1, and 0.1 units/ml recombinant LDH was prepared. 3-PG was added last at a final concentration of 2 mM to initiate the reaction. The decrease in autofluorescence (ex:340 nm, em:460 nm) from oxidation of NADH was measured as PGAM1 activity.

Detection of ferroptosis related phenotypes: The assays (FerroOrange, BDP 581/591 C11, ROS Assay Kit, JC-1 MitoMP Detection Kit and mtSOX Deep Red) were purchased from Dojindo Ltd. (Shanghai) Experiments were performed according to the manufacturer's instructions.
